# A Dynamic Epistemic Logic with Lying and Theory of Mind Limitations

**DOI:** 10.1007/s10849-026-09479-7

**Published:** 2026-08-11

**Authors:** Jakob Dirk Top, Rineke Verbrugge, Harmen de Weerd

**Affiliations:** Bernoulli Institute, University of Groningen, Nijenborgh 9, Groningen, 9747AG Groningen The Netherlands

**Keywords:** Theory of mind limits, Lying, Dynamic epistemic logic, Action models

## Abstract

*Theory of mind *(ToM) is the ability to attribute and reason about unobservable mental states of others, such as knowledge, beliefs, and intentions. ToM is needed in social interactions, negotiations, cooperation, and deception, but there is a limit to the recursive depth of ToM attributions humans can make. Researchers in epistemic logic classically assume that agents have common knowledge of honesty and perfect rationality, whereas humans lie and have ToM limitations. In our present work, we bridge this gap by developing a sound and strongly complete dynamic epistemic logic that models both lying and human ToM limitations. The design of our logic is inspired by findings from prior behavioural research. Our logic makes use of action models to model belief change caused by lying and other types of announcements. Using theoretical examples, we show how a listener deals with lies that she cannot understand due to her ToM limits.

## Introduction

The goal of this paper is to develop a dynamic logic that can model both lying announcements *and* human theory of mind limitations. Such a logic can be used to explain and make predictions about human decisions in behavioural tasks, and it can be deployed in computational models of human reasoning. Moreover, in its development, we identified some promising directions for future computational cognitive modelling and behavioural research.

In Section 1.1 we give an overview of relevant findings on theory of mind. In Section 1.2 we discuss lying and deception. Relevant research in formal logic is presented in Section 1.3. Lastly, in Section 1.4, we give a section overview of our paper.

### Theory of mind

*Theory of mind* (ToM) is the ability to attribute and reason about the unobservable mental content of others, such as knowledge, beliefs, and intentions (Premack & Woodruff, 1978; de Weerd et al., 2013). ToM can be used recursively. For example, if Amy knows that Ben knows that Amy knows that there will be a surprise party, then Amy is using second-order ToM, or ToM-2. Someone’s ToM order is the number of times they can switch mental (not visual) perspectives. A ToM-0 user can only reason about observable behaviour and world facts.

Switching perspectives is useful in many contexts. Higher orders of ToM improve (pro-)social behaviour (Liddle & Nettle, 2006; Stiller & Dunbar, 2007; Imuta et al., 2016), negotiation skills (de Weerd et al., 2015, 2017, 2022; Peterson et al., 2018; Lee et al., 2021), performance on strategic games (Nagel, 1995; Hedden & Zhang, 2002; Goodie et al., 2012; Verbrugge et al., 2018; Veltman et al., 2019), cooperation (Paal & Bereczkei, 2007; Etel & Slaughter, 2019), and deception capability, as well as the detection thereof (Wimmer & Perner, 1983; Talwar et al., 2007). Human ToM capabilities improve with age (Wimmer & Perner, 1983), and training, prompting, and feedback can also improve or accelerate ToM use (Verbrugge et al., 2018; Veltman et al., 2019; Arslan et al., 2020).

Most epistemic logics assume common knowledge of perfect rationality, in this context by employing S5 and/or having no limits on model height. In contrast, there are limits on the ToM orders that even adult humans can use, as shown in consistent mental attribution mistakes across a wide range of tasks (Kinderman et al., 1998; Keysar et al., 2003; Birch & Bloom, 2007; Bernstein et al., 2011; Devaine et al., 2014; Zhang et al., 2021; Minculescu et al., 2025). There are individual ToM limits that are task- and subject-dependent (Flobbe et al., 2008), but there is also some hard upper limit to the ToM orders any one human can use, caused not by biases like the curse of knowledge (Ghrear et al., 2016), but by limitations on working memory, mental processing speed, and size restrictions on the orbital prefrontal cortex, which have all been shown to correlate with mentalizing capability (Mutter et al., 2006; Wynn & Coolidge, 2009; Lin et al., 2010; Powell et al., 2010).

Perspective-taking is difficult, even if all other factors of a task are held constant. The experiments of Apperly et al. (2010), Meijering et al. (2013), and Bradford et al. (2018) show that participants are slower and more error-prone when reasoning about someone else than about themselves, about a simple exclusion rule, or about a mechanical balance scale. There seems to be a qualitative difference between reasoning about one’s own beliefs, versus reasoning about the beliefs of others, which plays a critical role in the development of our logic.

In fact, recursive belief attribution appears to be intractable (Zawidzki, 2013; van de Pol et al., 2018). Furthermore, ToM use may be limited by a serial processing bottleneck, which limits the amount of stored information we can process simultaneously (Verbrugge, 2009; Borst et al., 2010; Arslan et al., 2017a; Vogelzang et al., 2017).

To summarize, the number of times any one of us can recursively switch perspectives is finite, which is the key idea our logic is based upon. Based on the findings discussed in the present section, we propose three design criteria for our logic. First, agents (recursively) reason with specific ToM orders. Different agents may use different orders, and single agents may use several orders at once [see, e.g., de Weerd et al. (2013)]. Second, there is some global maximum ToM order beyond which no agent can reason. Third, the ToM order at which an agent reasons restricts the number of times it can switch perspectives between different agents.

### Lying and deception

Theory of mind can be used to deceive others (Byrne & Whiten, 1988; Braüner et al., 2020). There are many ways to define a lie (Meibauer, 2019; van Ditmarsch et al., 2020a), but for our purposes, someone is *lying* when they utter some sentence *p* even though they believe that ¬p, with the intention that someone else now believes that *p* (van Ditmarsch, 2014a). Lies can be pro-social, such as white lies, but they can also be harmful, such as scams. ToM is required for producing successful lies: The experiments in Talwar et al. (2007), Talwar and Lee (2008), and Evans and Lee (2013) show that children who are better at lying also perform better on story tasks that require ToM, and that spontaneous lying emerges between the ages of two and three years.

Humans deceive in many contexts (Rosenbaum et al., 2014). We keep lost items, don’t reveal undeserved windfalls, misreport self-reported outcomes, lie in sender-receiver games (Gneezy, 2005), and more. Lying habits are not homogeneous: Some people unconditionally tell the truth no matter the payoff, whereas others lie no matter what happens (Rosenbaum et al., 2014). Most people disapprove of lying, or at least believe that most others do (Bicchieri et al., 2023).

While there are a few ‘wizards of deception detection’ (O’Sullivan & Ekman, 2004), humans are generally poor at detecting deception (Granhag & Vrij, 2005). In fact, according to the Truth-Default Theory, people assume that others tell the truth by default (Levine, 2014). Based on prior behavioural research, Levine claims that ‘the possibility that a message might be deception often does not come to mind unless suspicion is actively triggered.’ According to this theory, most lies are detected after-the-fact based on confessions or new evidence. Outside of the lab, very few lies are detected through non-verbal behaviour. The work of (Yeter et al., 2024, 2025) shows that some lies are also detected through *semantic leakage*: Accidentally giving information that could not be known if one was telling the truth.

Based on the findings presented in this section, we propose two more design criteria for our logic. First, our logic should be capable of modelling public and private lying announcements. Second, as people can make announcements about higher-order ToM statements, it should be possible that an agent’s ToM order affects what it learns from statements that use more perspective switches than it is capable of.

### Related literature in formal logic

The present paper aims to bridge the gap between logics with ToM limits and similar restrictions on the one hand, and formal accounts of lying on the other. In this section, we give an overview of relevant approaches in formal logic, starting with logical accounts of ToM limitations.

#### Theory of mind limitations in logic

Perhaps the oldest work on logics with ToM-like limitations can be found in Halpern (1995), where $\mathcal {L}_n^k(\Phi )$ is the *n*-agent language of epistemic logic where the nesting depth of modal operators is at most *k*. As an example, $K_1K_1p$ is a formula in $\mathcal {L}_n^2(\Phi )$, while $K_1K_1K_1p$ is not. Contrary to the behavioural findings discussed in Section 1.1, *any* modal operator increases a formula’s depth, regardless of the number of perspective switches. This depth is called the *degree* in Blackburn et al. (2001), where the *height* of an epistemic model is defined by induction: The height of the root is 0, and the states of height n+1 are the immediate successors of states of height *n* that have not yet been assigned a height smaller than n+1.

Zhang et al. (2021) tasked participants to solve epistemic puzzles called ‘Aces and Eights’ at different ToM orders. The authors develop computational models that use dynamic epistemic logic, where the height of the epistemic model is bounded to represent the bounds on human ToM reasoning. Using their models, the authors estimate the prevalence of different ToM orders in their participant population, revealing that most participants use first-order ToM. Unlike the logic we create, Zhang and colleagues only model truthful public announcements.

Counting the *number* of nestings of any modal operators is insufficient for our purposes. We are also interested in *how* modal operators are nested. In Kaneko and Suzuki (2002), the *epistemic depth* of a formula is defined as a set of sequences of agents, where each sequence is a path through the formula’s syntactic tree. As an example, the formula $B_4(B_2B_1p\wedge B_3p)$ has epistemic depth $\{(4,2,1),(4,3)\}$. A language $\mathcal {P}_E$ is the subset of the language $\mathcal {P}$ restricted to those formulas that have an epistemic depth in *E*. However, it is not discussed which *E* should be chosen to reflect human behaviour. Furthermore, the logic is static: The beliefs of agents cannot change.

The first work on depth-bounded *dynamic* logic may be Arthaud and Rinard (2023). It describes a public announcement logic with special depth atoms, where $E_a^d$ means ‘agent *a* has exact depth *d*’, and $P_a^d$ means ‘agent *a* has at least depth *d*’. If an agent cannot understand an announcement because its depth is too low, then it does not know whether the announcement has occurred. The logic does not distinguish between self-directed and other-directed ToM, contrary to the empirical findings discussed in Section 1.1, and only truthful public announcements are modelled.

The logic we present in Top et al. (2024) *does* distinguish between switching and not switching perspectives, but it cannot be used to model deception, because it only models *truthful public announcements*. In our previous logic, an epistemic model is defined as an M=(S,R,V,T), where *S*, *R*, and *V* are the states, relations and valuations defined in the usual manner, while $T: S\rightarrow \mathcal {P}(A\times \mathbb {N})$ is a so-called ‘ToM map’ that assigns, to each state in *S*, a set of tuples consisting of an agent in *A* and a ToM order in $\mathbb {N}$. Public announcements eliminate the possibility that agents have certain ToM orders at each state, by removing tuples from the ToM map. Validity is defined on state-tuple pairs. For example, given an epistemic model *M*, a state *s*, an agent *i*, and her ToM order *l*, then for i≠j we have $M, (s,(i,l))\models K_j\varphi \Leftrightarrow M, (t,(j,l-1))\models \varphi$ for all (t,(j,l-1)) with $(s,t)\in R_j$ and (j,l-1)∈T(t). Here, by taking l-1, an agent *i* always attributes her own ToM order, minus one, to other agents *j*. This may contradict real life, as adults capable of second-order ToM may still sometimes attribute zero-order ToM to animals or very young children. Furthermore, in our previous logic we only define a syntax and a semantics. We do not provide an axiom system, and we do not show soundness or completeness. Nonetheless, in Top et al. (2024) we go beyond purely theoretical work by creating logic-based computational models and fitting them on the data of Zhang et al. (2021).

Before we discuss logics of lying, we briefly look at a few papers that are not directly related to our work, but do skirt topics similar to ToM limitations. Dégremont et al. (2014) define the ‘horizon’ of an agent *i* as all states agent *i* can reach by taking one step along one of its own relations, followed by any number of steps along any agent’s relations. The logic in Solaki (2022), like the one presented in this paper, is also inspired by experimental findings. There, the number of inference steps is restricted, as opposed to the number of modal operators. We are also aware of the large body of work on logics with resource-bounded agents, such as Alechina and Logan (2009), where agents need *time* to perform inferences, and Nguyen and Rakib (2023), where agents are resource-bounded and *probabilistic*. In the present paper we focus only on ToM limitations and deception, leaving bounds on inference steps, working memory limitations, and bounds on any other cognitive resources for future work. Similar to Top et al. (2024), we model ToM limitations by placing bounds on the structure of epistemic models, and by having an agent’s ToM affect how its beliefs are updated.

#### Logics of lying

One of the first formal models of lying may be O’Neill (2003). Here, $C_{ab}p$ is read as ‘agent *a* communicates to agent *b* that *p*’. A lie is defined as $Lie_{ab}p = C_{ab}p \wedge B_{a}\lnot p$, where $B_a\lnot p$ is read as ‘Agent *a* believes *p* is false’. A similar approach is found in Sakama et al. (2010) and Sakama et al. (2015), where an agent *a* is defined to lie to *b* that σ, if *a* utters σ to *b*, believes that $\lnot \sigma$, and intends for *b* to believe that σ. In their syntax: $LIE_{ab}(\sigma ){\mathop {=}\limits ^{def}}utter_{ab}(\sigma )\wedge B_a\lnot \sigma \wedge I_aB_b\sigma$.

These logics are *static*: They cannot model belief *change* caused by lies. Baltag and Smets (2008) use *action models* to *dynamically* model a ‘public successful lie’: If an agent *a* lies to agent *b*, agent *a* knows whether she is lying, while agent *b* does not know whether *a* is lying. The same type of action models are used in van Ditmarsch et al. (2012), which presents a complete axiomatization of a novel logic that uses action models to model public lies made by an external observer.

Not all lies are public, and external observers are not the only liars. In the influential work of van Ditmarsch (2014a, 2014b), several logics of lying are introduced, along with their axiomatizations. A distinction is made between public lies by an outside observer and private lies by a single agent. Three types of agents are defined: Credulous agents who believe any lie, skeptical agents who reject lies that are inconsistent with their beliefs, and belief revising agents who use plausibility models to revise their beliefs.

In work from the same author, it is shown that lying can also be modelled using arrow updates instead of action models (van Ditmarsch et al., 2020b). Action models have since been used to model lying and misinformation (Chow, 2020), how agents recover from false beliefs caused by lying (Li & van Eijck, 2022), and visual lies such as the ones that occur in magic tricks (Icard & Fervari, 2024).

All logics discussed in this section assume common knowledge of perfect rationality. This is in stark contrast to the human ToM limitations discussed in Section 1.1. In the present paper, we aim to fill this gap by proposing an action model logic with lying and ToM limitations. Our work is a continuation of Top et al. (2024), which proposes a semantics for ToM limitations without lying.

### Paper overview

In Section 2, we present the syntax and semantics of a logic with theory of mind, show its soundness and strong completeness, and give an example of a model in this logic. In Section 3, we add action models to our logic, and again show soundness and strong completeness. In Section 4, we cover limitations of our logics and discuss future work.

## A logic of theory of mind

In the current section we present our logic with theory of mind (ToM) limitations, which we will extend with action models in Section 3. Our formalization mainly draws inspiration from Baltag and Moss (2004), Baltag et al. (2023), Meyer and van der Hoek (1995), and van Ditmarsch et al. (2007), and often reuses their notational conventions. In Section 2.1, we present our language. Section 2.2 introduces a variation of Kripke models where the accessibility relations have ToM orders. Our semantics is defined in Section 2.3. Sections 2.4 and 2.5 showcase several axiom systems, including one for ToM and one for introspection. The soundness and strong completeness of our systems is shown in Section 2.6. Lastly, Section 2.7 describes two examples of Kripke models with ToM.

### Language

First, we define the language of theory of mind (ToM) without action operators, which we call $\mathcal {L}_{\text {ToM}}$.

#### Definition 1

(*Language of the logic of ToM*) Let *A* be a finite set of agents, *P* a countable set of propositional atoms, and $L {:}{=} \{i\in \mathbb {N}_0\,|\,i\le \text {Max}\}$ for fixed $\text {Max}\in \mathbb {N}_0$ (here, $0\in \mathbb {N}_0$). Then, the language of the logic of ToM is defined using the following BNF [based on van Ditmarsch et al. (2007); Meyer and van der Hoek (1995)]:

$$\begin{aligned} \mathcal {L}_{\text {ToM}}(A, P, \text {Max}) \ni \varphi {:}{:}{=} \quad p\quad |\quad \lnot \varphi \quad |\quad (\varphi \wedge \varphi )\quad |\quad B_i^l\varphi \quad \end{aligned}$$

with i∈A, p∈P, and l∈L.

Here, we follow the notation and conventions of van Ditmarsch et al. (2007). The usual abbreviations are used for ∨, →, ↔, ⊥, and ⊤. For $\lnot B_i^l\lnot \varphi$ we use $\hat{B}_i^l\varphi$. By abbreviation we define $B_i\varphi = \bigwedge _{l\in L}B_i^l\varphi$. A formula $B_i\varphi$ holds in *s* iff φ holds in all worlds that agent *i* can reach from *s* in one step, using *any* of her ToM orders. For $\lnot B_i\lnot \varphi$ we use $\hat{B}_i\varphi$. A formula $B_i\varphi$ is read as ‘agent *i* believes that φ’, and $\hat{B}_i\varphi$ is read as ‘agent *i* considers it possible that φ’. Furthermore, $B_i^l\varphi$ is read as ‘agent *i*, at ToM order *l*, believes that φ’ and $\hat{B}_i^l\varphi$ is read as ‘agent *i*, at ToM order *l*, considers it possible that φ’.

We use *L* instead of $\mathbb {N}_0$ to ensure a *finite* number of preconditions for each action point in our action models, which will be introduced in Definition 11. In Section 1.1, we show that there are limits to the ToM orders that even adult humans use: A conservative estimate such as $\text {Max} = 42$ should be sufficient to model any human interaction.

### Theory of mind-enhanced models

Next, we define a variation of Kripke models we call *ToM-enhanced (ToMe) models*, which encode not only each agent’s beliefs, but also each agent’s beliefs at each ToM order that that agent might use. Instead of having a relation function for each agent, we have a relation function for each combination of an agent and a ToM order in *L*.

#### Definition 2

(*Models with theory of mind*) A ToM-enhanced (ToMe) model M=(S,R,V) consists of a non-empty domain of states *S*, a finite accessibility function $R : (A \times L) \rightarrow \mathcal {P}(S \times S)$, and a valuation $V : P \rightarrow \mathcal {P}(S)$. For s∈S, (*M*, *s*) is an *epistemic state*. We may omit the parentheses when no ambiguity can arise.

Because $B_i\varphi$ is defined as the conjunction over $B_i^l\varphi$ for all ToM orders *l*, it is possible that an agent knows more if she only takes her higher ToM orders into account. For example, if $B_a^0 p$ is false, and $B_a^1 p$ is true, then $B_a p$ will also be false. One way of making $B_a p$ true is by eliminating all of agent *a*’s ToM-0 relations, making $B_a^0 p$ vacuously true. This example shows that an agent should not always use *all* ToM orders it has access to.

### Semantics

We mostly follow the usual semantics of epistemic logic.

#### Definition 3

(*Semantics of the logic of ToM*) Given i∈A, p∈P, l∈L, let M=(S,R,V) be a ToMe model. Then we define:

$$\begin{array}{lll} M,s\models p& \text {iff}& s\in V(p)\\ M,s\models \lnot \varphi & \text {iff}& M,s\not \models \varphi \\ M,s\models \varphi \wedge \psi & \text {iff}& M,s\models \varphi \text { and }M,s\models \psi \\ M,s\models B_i^l\varphi & \text {iff}& M,t\models \varphi \text { for all }t\in S\text { with }(s,t)\in R(i,l)\\ \end{array}$$

Intuitively, most humans do not explicitly reason about which exact numeric ToM order others use, but rather just *use* their ToM, with the possible exception of ToM researchers, and professional spies. Wondering what someone else is thinking is common, whereas wondering whether someone else is reasoning at ToM-1 or reasoning at ToM-2 seems rare. Because of this, we wish to avoid being able to construct sentences such as $B_i^lE_a^d$ in our language, where $E_a^d$ would mean ‘agent *a* has exact depth *d*’. For these reasons we opt not to include explicit atoms for agents’ ToM orders, unlike Arthaud and Rinard (2023), where such atoms exist.

### The axiom system ToMe

The axiom system for our most basic logic is in direct parallel to the well-known axiom systems for modal and epistemic logics.

#### Definition 4

(*Axioms and rules of ToMe*)

$$\begin{array}{|l|l|l|} \hline \text {A1}& \text {Prop. Taut.}& \text {All instantiations of propositional tautologies}\\ \hline \text {A2}& \text {Distribution}& (B_i^l\varphi \wedge B_i^l(\varphi \rightarrow \psi ))\rightarrow B_i^l\psi \\ \hline \end{array}$$

The rules are as follows. R2 can only be used on theorems.

$$\begin{array}{ll} \text {R1 (Modus Ponens):}& \text {From }\varphi \text { and } \varphi \rightarrow \psi \text {, infer }\psi \\ \text {R2 (Necessitation):}& \text {From }\varphi \text {, infer }B_i^l\varphi \\ \end{array}$$

Here, *i* ranges over all i∈A, and *l* ranges over all l∈L.

### Additional axioms and model properties

This section shows several axioms and model properties that can be added to ToMe.

#### Axioms and model properties of system ToM

An important property of theory of mind is that a ToM-*m* user cannot attribute ToM-*l* to others for l≥m. Also, a ToM-*m* user cannot attribute ToM-*l* to herself for l>m: Otherwise, as an example, we could construct a model with relations $(s_1,s_2)\in R(a,0)$, $(s_2,s_3)\in R(a,2)$, and $(s_3,s_4)\in R(b,1)$, allowing agent *a* at ToM-0 to indirectly attribute ToM-1 to agent *b*. Unlike in our previous work (Top et al., 2024), where agents use a fixed ToM order and always attribute their own order, minus one, to other agents, we follow de Weerd et al. (2013) in letting an agent reason at any number of ToM orders, provided that each of these ToM orders is at least zero, and at most $\text {Max}$.

This yields the following two model properties:

##### Definition 5

(*Theory of mind models*) We define two more model properties:

- *Downward-ToM-Other*: There are no $(s_1, s_2) \in R(i, m)$ and $(s_2, s_3) \in R(j, l)$ with i≠j and l≥m.
- *Downward-ToM-Self*: There are no $(s_1, s_2) \in R(i, m)$ and $(s_2, s_3) \in R(i, l)$ with l>m.

A ToMe model with both properties is said to be a ToM model.

Note here that in the second property, the agent is *i* in both relations. This class of models is intended to have the minimum properties required to model theory of mind, so despite system ToM having more model restrictions than ToMe, its name is shorter. For ToM we extend the axiom system of ToMe with two additional axioms:

##### Definition 6

(*Axioms of ToM*)

$$\begin{array}{|l|l|l|} \hline \text {DTO}& \text {Downward-ToM-Other}& B_i^mB_j^l\bot \text { for }l\ge m,i\ne j\\ \hline \text {DTS}& \text {Downward-ToM-Self}& B_i^mB_i^l\bot \text { for }l>m\\ \hline \end{array}$$

Note that in DTS, the agent is *i* for both belief operators. Also note that, if we replace l>m in axiom DTS with l≥m, we disallow agents from attributing their own ToM order to themselves. This more closely resembles Arthaud and Rinard (2023), but is not in line with our own intentions: We assume there is a qualitative difference between reasoning about one’s own beliefs and someone else’s beliefs, as supported by works such as Nichols and Stich (2002) and Bradford et al. (2018).

The properties of ToM ensure that any agent *i* at ToM order *m* is confused about the beliefs of any different agent *j* at ToM order *l* with l≥m: If there is an incoming *i*, *m*-relation at a state *s*, then there cannot be an outgoing *j*, *l*-relation at the same state *s* for l≥m and i≠j. Agents with no outgoing relations are commonly treated as being ‘crazy’ (Aucher, 2008; van Ditmarsch & Kooi, 2008). In the present paper, we use the interpretation that an agent with no outgoing relations at a world *s* simply does not *exist* at that world *s* as an agent (or, at least, its mental states do not exist, as ToM concerns reasoning about mental states), and therefore vacuously believes everything. By extension, an agent reasoning about a non-existent agent is confused, and believes that a non-existent agent believes in everything, including contradictions.

#### Axioms and model properties of system ToM45 and related systems

As a reminder, one of our goals is modelling *lies*. Therefore, we do not use reflexivity, as an agent’s beliefs may be false after being lied to. In addition, the property Downward-ToM-Other prohibits us from using seriality. Consider a state $s_1$ with an incoming ToM-0 relation for some agent *i*, that is, $(t,s_1)\in R(i,0)$ for some *t*. Then, because of Downward-ToM-Other, there cannot be a state $s_2$ such that $(s_1,s_2)\in R(j,l)$ for i≠j and l≥0, which is *all* l∈L. Therefore, at $s_1$, there are no outgoing relations for any agents other than *i*: A ToM-0 agent does not consider it possible that any other agent considers anything to be possible: It is unable to model the beliefs of others (de Weerd et al., 2013), and cannot do any perspective switches. Instead, we use positive introspection and negative introspection by including transitivity and euclidicity. The latter imposes a ‘weaker kind of seriality’ on our models, where a state with an incoming agent *a* relation must also have one or more outgoing agent *a* relations, due to euclidicity.

As relations now have ToM orders, we specify how transitivity and euclidicity interact with ToM orders. The properties are as follows:

##### Definition 7

(*ToM45 models*)

- *ToM-Transitive*: If $(s_1,s_2)\in R(i,l)$ and $(s_2,s_3)\in R(i,m)$ with l≥m, then also $(s_1,s_3)\in R(i,m)$.
- *ToM-Euclidean*: If $(s_1,s_2)\in R(i,l)$ and $(s_1,s_3)\in R(i,m)$ with l≥m, then also $(s_2,s_3)\in R(i,m)$.

A ToM model that is ToM-Transitive is called a ToM4 model. A ToM model that is ToM-Euclidean is called a ToM5 model. A ToM model that has both properties is called a ToM45 model.

**Fig. 1:**
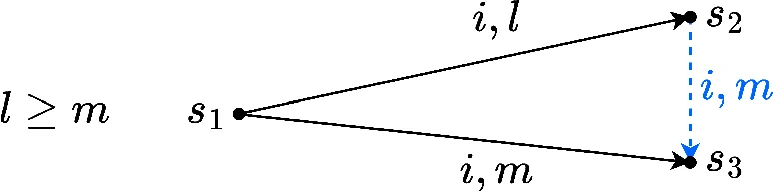
ToM-Euclidean: If $(s_1,s_2)\in R(i,l)$ and $(s_1,s_3)\in R(i,m)$ with l≥m, then also .

As an example, ToM-Euclidicity is graphically shown in Fig. 1.

For ToM45 we extend the axiom system of ToM with two additional axioms:

##### Definition 8

(*Axioms of ToM45*)

$$\begin{array}{|l|l|l|} \hline A4& \text {ToM-Transitive} & B_i^m\varphi \rightarrow B_i^lB_i^m\varphi \text { for }l\ge m \\ \hline A5& \text {ToM-Euclidean} & \lnot B_i^m\varphi \rightarrow B_i^l\lnot B_i^m\varphi \text { for }l\ge m \\ \hline \end{array}$$

An interesting property of ToM4 models is that their height is at most $\text {Max} + 1$. Height is defined in Definition 9, and we prove this property in Proposition 1.

##### Definition 9

(*Model height*) Following Blackburn et al. (2001), the *height* of a model M=(S,R,V), with some root s∈S, is defined by induction: The height of the root is 0, and the states of height n+1 are the immediate successors of states of height *n* that have not yet been assigned a height smaller than n+1. The height of a model *M* is the maximum of the height of its states. If there is no maximum, the height is infinite.

##### Proposition 1

The height of ToM4 models is at most $\text {Max} + 1$.

##### Proof

To prove that the height of ToM4 models is at most Max +1, we first prove the claim that “in ToM4 models, for non-root states of height *n*, the ToM order of incoming relations is at most Max $+\ 1\ -\ n$”. Let M=(S,R,V) be a ToM4 model. We prove our claim by induction over *n*:

- **Base case** (n = 1) This follows immediately from the definitions of *R* and *L*.
- **Inductive Hypothesis** For each non-root state s∈S with a height *k* such that k≤n for some arbitrary *n* with n≥1, assume that the ToM orders of the incoming relations of *s* are at most Max $+\ 1\ -\ k$.
- **Inductive Step** $(n + 1\text { for }n\ge 1)$ Take an arbitrary non-root state *u* with height n+1. We have to show that the ToM order of the incoming relations of *u* are at most $\text {Max}\ +\ 1\ -\ (n\ +\ 1)$, or Max -n. From the definition of height there must be a state *t* with height *n* such that (t,u)∈R(j,m) for some *j*, *m*. What we now have to show is that $m\le \text {Max} - n$. Since *t* is a non-root state, there must also exist some state *s* with height n-1 such that (s,t)∈R(i,l) for some *i*, *l*. By the inductive hypothesis, the ToM order of the incoming relations of *t* have a height at most Max $+\ 1\ -\ n$. Therefore $l \le \text {Max} + 1 - n$. Now there are two possibilities:
  - **Case 1.** i=j. From this, (s,t)∈R(i,l), (t,u)∈R(j,m), and Downward-ToM-Self it follows that l≥m. From ToM-Transitivity it now also follows that (s,u)∈R(i,m). But *s* has height n-1 and *u* is an immediate successor of *s*, so *u* cannot have height n+1, leading to a contradiction. Therefore it must be the case that i≠j.
  - **Case 2.** i≠j. From this, (s,t)∈R(i,l), (t,u)∈R(j,m), and Downward-ToM-Other it follows that l>m. From the latter and $l \le \text {Max} + 1 - n$ it follows that $m\le \text {Max} - n$, as required.

Now that we have shown by induction that “in ToM4 models, for non-root states of height *n*, the ToM order of incoming relations is at most Max $+\ 1\ -\ n$”, we can proceed to show that the height of ToM4 models is at most $\text {Max} + 1$. Let M=(S,R,V) be a ToM4 model, and suppose a t∈S with height $\text {Max} + 1$. Then the ToM order of the incoming relations of *t* can be at most 0. By the definition of height, there must be some s∈S with height $\text {Max}$ such that (s,t)∈R(i,0) for some *i*. Towards a contradiction, assume that there exists some u∈S such that *u* has height $\text {Max} + 2$ and (t,u)∈R(j,m). Now there are two possibilities:

- **Case 1.** i=j. From this, (s,t)∈R(i,0), (t,u)∈R(j,m), and Downward-ToM-Self it follows that 0≥m and therefore m=0. From ToM-Transitivity, it now also follows that (s,u)∈R(i,0). But *s* has height Max and *u* is an immediate successor of *s*, so *u* cannot have height $\text {Max} + 2$, leading to a contradiction.
- **Case 2.** i≠j. From this, (s,t)∈R(i,0), (t,u)∈R(j,m) and Downward-ToM-Other, it follows that 0>m, which yields a contradiction.

Since both cases yield a contradiction, there cannot exist a u∈S such that *u* has height $\text {Max} + 2$. From this we conclude that the height of ToM4 models can be at most $\text {Max} + 1$. □

### Soundness and strong completeness of systems ToMe, ToM, and ToM45

Soundness and strong completeness of ToMe follow directly from the fact that ToMe is a multi-agent *K* logic (Blackburn et al., 2001). In fact, it is easy to see that ToMe can be expressed in multi-agent *K* logic by defining a bijection $(A_{\text {ToMe}}\times L)\rightarrow A_{K}$, where $A_{\text {ToMe}}$ is the set of agents in ToMe, *L* is the set of possible ToM orders, and $A_{K}$ is the set of agents in multi-agent *K* logic such that $|A_{K}| = |A_{\text {ToMe}}|\cdot |L|$, where |*A*| denotes the cardinality of *A*. From this it also follows that ToMe is a normal modal logic [see Blackburn et al. (2001)], allowing us to use the standard completeness-by-canonicity argument [see, e.g, de Jongh and Veltman (1999), Blackburn et al. (2001), or van Ditmarsch et al. (2007)]. Completeness of axioms DTO, DTS, A4, and A5 can be shown by showing that if the axiom is added to the axiom system of ToMe, then the canonical model has the corresponding model property. Soundness can be shown by showing that our axioms are valid in frames with the corresponding model properties, and by showing that our rules preserve validity. We skip most of the soundness and completeness proofs for ToMe, because these are near-identical to the usual proofs for system *K*. Our canonical model definition somewhat deviates from the usual definition, and can be found in Definition 10.

Our logics of interest are ToM and ToM45, as they have the properties of theory of mind and of introspection. The proofs for soundness and strong completeness of axioms A4 and A5 are near-identical to the usual proofs for the transitivity and euclidicity axioms, so we skip them. Axioms DTO and DTS are novel, so we show the soundness and strong completeness proofs for DTO in Lemma 1 and Lemma 2, leaving the highly similar proofs for DTS to the reader. We then proceed to show soundness and strong completeness for axiom systems ToMe, ToM, and ToM45 in Theorem 1.

#### Lemma 1

Axiom DTO is valid in frames with the property *Downward-ToM-Other*.

#### Proof

Consider a frame *F* with the property Downward-ToM-Other, i.e. ‘There are no $(s_1, s_2) \in R(i, m)$ and $(s_2, s_3) \in R(j, l)$ with i≠j and l≥m.’ Assume an arbitrary $s_1\in S$, with given m∈L. We have two cases. One: There are no $s_2\in S$ with $(s_1,s_2)\in R(i,m)$. In this case $B_i^mB_i^l\bot$ is vacuously true. Two: There *are* $s_2\in S$ with $(s_1,s_2)\in R(i,m)$, in which case for each of these $s_2$ there is no $(s_2,s_3)\in R(j,l)$ with l≥m and i≠j, because otherwise we violate the property Downward-ToM-Other. Because of this, at each of these $s_2$, we vacuously have $B_j^l\bot$ for any *j*, *l* with l≥m and i≠j, so at $s_1$ we have $B_i^mB_j^l\bot$. As $s_1$ was chosen arbitrarily we have $F\models B_i^mB_j^l\bot$. □

Next, we include our canonical model definition, as it is used in the proof for strong completeness of DTO. We assume that the reader is familiar with maximally consistent sets and the properties thereof, and we assume that the reader is familiar with the standard completeness-by-canonicity argument [for reference, see de Jongh and Veltman (1999), Blackburn et al. (2001), or van Ditmarsch et al. (2007)].^1^

#### Definition 10

The canonical model $M^c=(S^c,R^c,V^c)$ is defined with

$$ \begin{array}{lllll} & S^c& =& \{s_\Theta |\Theta \text { is a maximally consistent set}\}& \\ \text {For }s_\Theta \in S^{c} :& s_\Theta \in V^c(p) & \text {iff } & p\in \Theta & \\ & (s_\Theta ,s_\Psi )\in R^c(i,l)& \text {iff }& \text {for all }\varphi \text {, if }B_i^l\varphi \in \Theta \text {, then }\varphi \in \Psi & \\ \end{array} $$

What follows is the strong completeness proof for axiom DTO.

#### Lemma 2

If axiom DTO is added to the axiom system of ToMe, then the canonical model has the property Downward-ToM-Other.

#### Proof

First, rewrite Downward-ToM-Other to ‘If there is an $(s_1,s_2)\in R(i,m)$, then there are no $(s_2,s_3)\in R(j,l)$ with $i\ne j,\ l\ge m$’.

Assume DTO is added to our axiom system, and assume that there is an $(s_\Theta ,s_\Psi )\in R^c(i,m)$. We must show that there are no $(s_\Psi ,s_\Omega )\in R^c(j,l)$ with $i\ne j,\ l\ge m$. We know $B_i^mB_j^l\bot \in \Theta$ for $l\ge m,\ i\ne j$, due to each maximally consistent set containing all theorems. Since there is an $(s_\Theta ,s_\Psi )\in R^c(i,m)$, from the definition of our canonical model, we know that ‘for all φ, if $B_i^m\varphi \in \Theta$, then $\varphi \in \Psi$’. Since $B_i^mB_j^l\bot \in \Theta$, also $B_j^l\bot \in \Psi$ for all l≥m, j≠i. Now suppose for fixed *j*, *l* that there is some $(s_\Psi ,s_\Omega )\in R^c(j,l)$ with $i\ne j,\ l\ge m$. Then, from the definition of the canonical model, it must be the case that $\bot \in \Omega$, from which we have that Ω is inconsistent, yielding a contradiction. So there are no $(s_\Psi ,s_\Omega )\in R^c(j,l)$ with $i\ne j,\ l\ge m$, as required. □

Now, we can show soundness and strong completeness.

#### Theorem 1

The axiom systems ToMe, ToM, and ToM45 are sound and strongly complete with respect to their semantics.

#### Proof

Soundness and strong completeness of ToMe follow directly from it being a multi-agent *K* logic. Soundness of ToM follows from Lemma 1 and a similar proof for DTS. Strong completeness of ToM follows from Lemma 2 and a similar proof for DTS. Extending soundness and strong completeness from ToM to ToM45 is done by adding proofs for transitivity and euclidicity, which are near-identical to their usual proofs. □

Our axioms are not only sound, but also characterize the corresponding classes of frames (see de Jongh & Veltman, 1999; van Benthem et al., 2003). That is, a class of frames has some model property if and only if the corresponding axiom is valid on that class of frames. To obtain these proofs we only need an additional proof for the right-to-left direction of the soundness proof of each axiom. These proofs are left as an exercise for the reader.

### Example models

In the present section, we show two examples of ToM models. In Section 2.7.1, we show one way to transform a regular epistemic model into a ToM model, whereas in Section 2.7.2, we show an example of decreasing ToM orders.

#### An example of a model with theory of mind

**Fig. 2:**
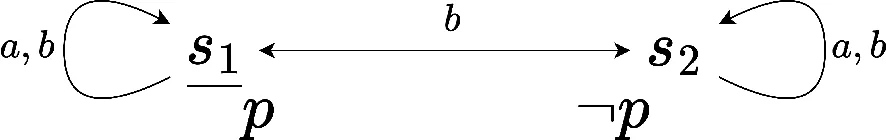
A simple model where *a* knows that *p*, and *b* does not (and this is common knowledge). The actual state has been underlined.

In this section we take a model in regular epistemic logic, and show what such a model would look like in ToM45. Consider an epistemic state $M, s_1$ with $s_1\in S$ such that M=(S,R,V) with states $S = \{s_1,s_2\}$, relations $R(a) = \{(s_1,s_1),(s_2,s_2)\}$ and $R(b) = \{(s_1,s_1),(s_1,s_2),(s_2,s_1),(s_2,s_2)\}$, and valuation $V(p) = \{s_1\}$, where $A=\{a,b\}$ and $P = \{p\}$. The corresponding graph can be found in Fig. 2.

Here, agent *b* does not know whether *p* is true, whereas agent *a* does. Now we add ToM orders. Because of the model property Downward-ToM-Other, we cannot give a single state reflexive relations for more than one agent.

**Fig. 3:**
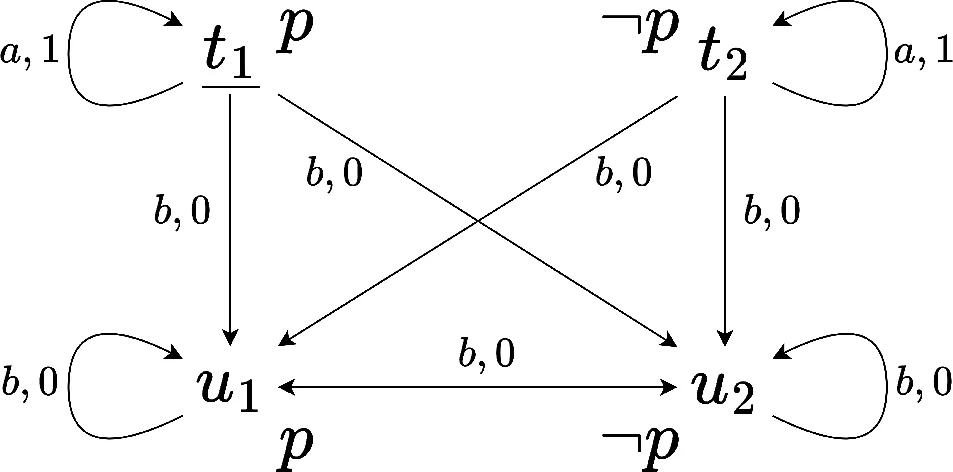
A simple model where *a* knows whether *p*, and *b* does not. Agent *a* uses ToM-1, and agent *b* uses ToM-0. The actual state is underlined. Being ToM-0, agent *b* is confused about agent *a*.

For the purposes of this example, let us suppose agent *a* is using first-order theory of mind (ToM-1), and agent *b* is using zero-order theory of mind (ToM-0). Furthermore, we assume agent *a* has no zero-order accessibility relations. We propose the epistemic state $M, t_1$ with $t_1\in S$, $A=\{a,b\}$, $P = \{p\}$ and $L=\{0,1\}$, where M=(S,R,V) with states $S = \{t_1,t_2,u_1,u_2\}$, relations $R(a,1) = \{(t_1,t_1),(t_2,t_2)\}$ and $R(b,0) = \{(t_1,u_1),(t_1,u_2),(t_2,u_1),(t_2,u_2),(u_1,u_1),(u_1,u_2),(u_2,u_1),(u_2,u_2)\}$, and valuation $V(p) = \{t_1,u_1\}$. The corresponding graph can be found in Fig. 3.

Note that this model is ToM45: It is ToM-Transitive and ToM-Euclidean. Because of Downward-ToM-Other, we cannot have an infinite sequence of relations that alternates between agents. However, we do have infinite sequences of relations with a *finite* number of alternations between agents, such as $(t_1,t_1)\in R(a,1), (t_1, u_1)\in R(b,0), (u_1,u_1)\in R(b,0), (u_1,u_1)\in R(b,0), \ldots$

**Fig. 4:**
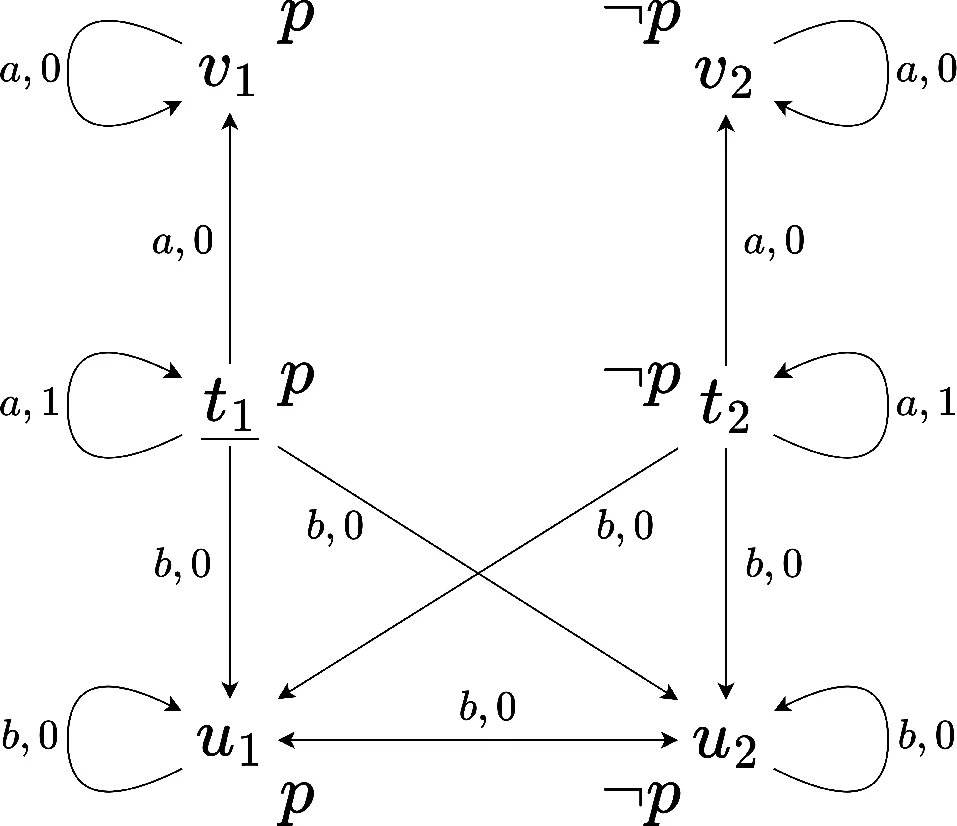
A simple model where *a* knows whether *p*, and *b* does not. Agent *a* uses both ToM-0 and ToM-1, and agent *b* uses ToM-0. The actual state is underlined. Being ToM-0, agent *b* is confused about agent *a*.

Alternatively, we can also construct a model in which agent *a* uses both ToM-0 and ToM-1, which is found in Fig. 4. In the model in Fig. 3, $M,t_1\models B_a\lnot B_b\bot$ holds, whereas in the model in Fig. 4, it does not.

#### An example of decreasing theory of mind orders

**Fig. 5:**
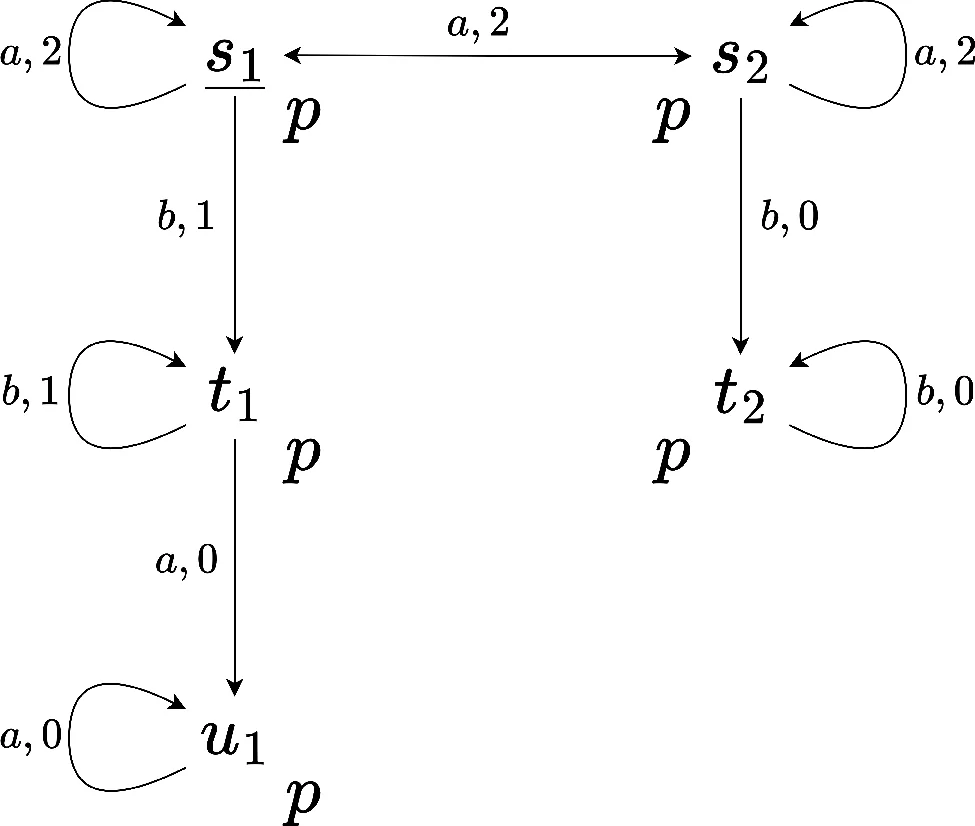
A model where in state $s_1$, agent *a*, at ToM order 2, considers it possible that *b* at ToM order 1 believes that *a* at ToM order 0 believes that *p*. In the same state $s_1$, agent *a* at ToM order 2 considers it possible that *b* at ToM order 0 believes that *p*.

Next, we explore the role of decreasing ToM orders. We show an example of a model where agent *a* is uncertain about the ToM order of agent *b*. Consider the epistemic state $M,s_1$ such that M=(S,R,V) with states $S = \{s_1, s_2, t_1, t_2, u_1\}$, relations $R(a,2) = \{(s_1,s_1),(s_1,s_2),(s_2,s_1),(s_2,s_2)\}$, $R(a,0) = \{(t_1,u_1),(u_1,u_1)\}$, $R(b,1)=\{(s_1,t_1),(t_1,t_1)\}$, and $R(b,0)=\{(s_2,t_2),(t_2,t_2)\}$ and valuation V(p)=S. The model can be found in Fig. 5.

In Fig. 5, in state $s_1$, agent *a* is uncertain about the ToM order of agent *b*. That is, she does not know whether agent *b* is confused and believes that agent *a* herself believes in contradictions. In epistemic logic, ‘not knowing whether φ’ is usually expressed as $\lnot K\varphi \wedge \lnot K\lnot \varphi$, so agent *a*’s uncertainty about *b*’s confusion can be expressed as $\lnot B_aB_bB_a\bot \ \wedge \ \lnot B_a\lnot B_bB_a\bot$. This formula is read as ‘agent *a* does not know whether *b* believes that *a* believes in the contradiction,’ and it is true in state $s_1$.

## Adding actions to logics of theory of mind

In the present section we introduce action models to ToMe. The resulting logic we will call ToMea, where the *a* signifies *actions*. Adding actions to ToM or ToM45 yields ToMa and ToM45a, respectively. Our methods are analogous to the ones employed in van Ditmarsch et al. (2007) and van Ditmarsch (2014a), with the crucial difference that our system adds theory of mind (ToM) orders as well as ToM limitations. Where possible, we employ the phrasing used in the aforementioned two works.

### Action models

Our action models are based on the work of van Ditmarsch et al. (2007) and are defined as follows. Note that our action models are not S5, as we use beliefs, and not knowledge.

#### Definition 11

(*Action Models*) Let $\mathcal {L}$ be *any* logical language for given parameters agents *A*, atoms *P*, and ToM orders *L*. An action model $\textbf{M}$ is a structure $(\textbf{S}, \textbf{R}, \textbf{pre})$ such that $\textbf{S}$ is a domain of *action points*, such that for each i∈A, $\textbf{R}(i)$ is a relation on $\textbf{S}$, and such that $\textbf{pre} : (\textbf{S}\times A\times L) \rightarrow \mathcal {L}$ is a preconditions function that assigns a *precondition* $\textbf{pre}(\textbf{s}) \in \mathcal {L}$ to each combination of an action point $\textbf{s}\in \textbf{S}$, an agent i∈A, and a ToM order l∈L. A *pointed action model* is a structure $(\textbf{M},\textbf{s})$ with $\textbf{s}\in \textbf{S}$. We may omit the parentheses when no ambiguity can arise.

Note here that relations in action models, unlike those in our ToM models, do not have ToM orders associated with them. While including them might increase the system’s expressive power, we opt to omit them to keep the system closer to existing systems with action models and therefore easier to understand. Instead, we modify the semantics of action models in order to handle ToM orders. Action models such that for i∈A and l∈L, $\textbf{R}(i,l)$ is a relation on $\textbf{S}$, are left as an avenue for future work. For such a system, our soundness and strong completeness proofs (Theorem 2 and Theorem 3) still hold under the perturbation of replacing $\textbf{R}(i)$ with $\textbf{R}(i,l)$.^2^

Unlike in the usual treatment of action models, we assign a precondition to each action point for each agent and each ToM order. This allows us to let agents at different ToM orders perceive the precondition of the same action point in a different manner. For example, when announcing $B_bB_ap$, agent *a* with second- or higher-order ToM may perceive the announcement as $B_bB_ap$, as she can do the two perspective switches required to understand the formula (from *a* to *b*, then back from *b* to *a*). For the same action point, we can assign ⊤ as precondition for agent *a* at ToM orders zero and one, effectively making her ‘ignore’ the precondition if she does not have the ToM order required to understand $B_bB_ap$. Alternatively, we could follow Minculescu (2024) by having agents cut belief operators from formulas that they do not understand. In this case, agent *a* at first- and zero-order ToM would ‘cut’ the $B_b$ operator and use precondition $B_ap$ instead. This corresponds to the finding that children who fail to use second-order ToM tend to revert to first-order ToM use, instead of choosing randomly (Arslan et al., 2017b).

### Language

To include action models, we modify the syntax presented in Definition 1. Our language is based on van Ditmarsch et al. (2007).

#### Definition 12

(*Language of the logic of ToM with action models*) Let *A* be a finite set of agents, *P* a countable set of propositional atoms, and $L {:}{=} \{i\in \mathbb {N}_0\,|\,i\le \text {Max}\}$ for fixed $\text {Max}\in \mathbb {N}_0$ (with $0\in \mathbb {N}_0$). The language of the logic of ToM with action models comprises the formulas $\varphi \in \mathcal {L}_{\text {ToMa}}(A, P, \text {Max})$, defined as follows:

$$\begin{array}{lll} \mathcal {L}_{\text {ToMa}}(A, P, \text {Max}) \ni \varphi & {:}{:}{=} & \ p\ |\ \lnot \varphi \ |\ (\varphi \wedge \varphi )\ |\ B_i^l\varphi \ |\ [\textbf{M},\textbf{s}]\varphi \\ \end{array} $$

with i∈A, p∈P, l∈L, and $(\textbf{M},\textbf{s})$ a pointed action model with finite domain $\textbf{S}$ such that for all $\textbf{t}\in \textbf{S}$, the precondition $\textbf{pre}(\textbf{t})$ is a finite $\mathcal {L}_{\text {ToMa}}(A, P, \text {Max})$-formula that has already been constructed in a previous step of the inductive definition of the language.

By abbreviation, we define $[\alpha \cup \beta ]\varphi = [\alpha ]\varphi \wedge [\beta ]\varphi$ (non-deterministic choice) and $B_i\varphi = \bigwedge _{l\in L}B_i^l\varphi$ (belief over all ToM orders of an agent). The usual abbreviations are used for ∨, →, ↔, ⊥, and ⊤. For $\lnot B_i^l\lnot \varphi$ we use $\hat{B}_i^l\varphi$, and for $\lnot B_i\lnot \varphi$ we use $\hat{B}_i\varphi$.

### Semantics

Our semantics uses the notation of van Ditmarsch et al. (2007). However, we depart from van Ditmarsch et al. (2007) and other semantics of action models by removing relations instead of removing states, which is more akin to arrow update logic (Kooi & Renne, 2011). Furthermore, our new precondition function includes agents and ToM orders as well.

#### Definition 13

(*Semantics of the logic of ToM with actions*) Given i∈A, p∈P, and l∈L, let M=(S,R,V) be a ToMe model, and let $\textbf{M} = (\textbf{S},\textbf{R},\textbf{pre})$ be an action model. Our semantics is as in Definition 3 with the following addition:

$$\begin{array}{lll} M,s\models [\textbf{M},\textbf{s}]\varphi & \text {iff}& (M\otimes \textbf{M}, (s,\textbf{s}))\models \varphi \\ \end{array}$$

In the interpretation of action models, $$M'=(M\otimes \textbf{M})$$ is a restricted modal product of an epistemic model and an action model, defined as $M^{\prime }=\left(S^{\prime },R^{\prime },V^{\prime }\right)$ with

Note that we do not remove any states. It is possible that no agent considers the actual state to be possible: For example, when both agents use ToM-1, both agents only consider it possible that the other agent uses ToM-0, so the true state, where both agents use ToM-1, is not considered possible by either agent.^3^

### Two examples of actions in models with theory of mind

In the present section, we show some examples of applying action models to epistemic models within our system.

#### An example of deception in a model with theory of mind

In this section, we show an example of applying a deceptive action to a model in our system. Consider once again the epistemic state $M, t_1$ shown in Fig. 3 in Section 2.7.1.

**Fig. 6:**
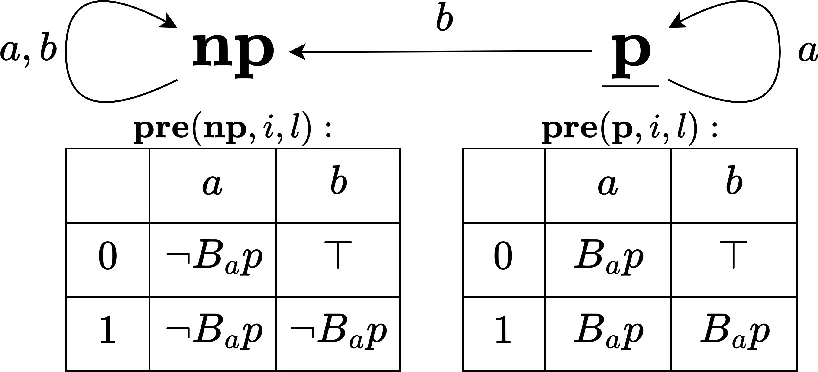
A pointed action where agent *a* lies to agent *b* that $\lnot B_a p$. The actual action has been underlined.

We resolve an action that models agent *a* lying to agent *b* that she does not believe that *p*. This action is based on those in van Ditmarsch (2014a). Agent *b* believes *a* is speaking truthfully. The model is a pointed action $(\textbf{M}, \textbf{p})$ with $\textbf{p}\in \textbf{S}$ and $\textbf{M} = (\textbf{S},\textbf{R},\textbf{pre})$ with action points $\textbf{S} = \{\textbf{np},\textbf{p}\}$, relations $\textbf{R}(a) = \{(\textbf{np},\textbf{np}),(\textbf{p},\textbf{p})\}$ and $\textbf{R}(b) = \{(\textbf{np},\textbf{np}),(\textbf{p},\textbf{np})\}$, and preconditions $\textbf{pre}(\textbf{np},b,0) = \textbf{pre}(\textbf{p},b,0) = \top$, while for all other *i*, *l* we have that $\textbf{pre}(\textbf{np},i,l) = \lnot B_ap$ and $\textbf{pre}(\textbf{p},i,l) = B_ap$. When using zero-order ToM, agent *b* cannot perform the perspective switch required to understand $B_ap$ or $\lnot B_ap$, so in this example we assign her the precondition ⊤, effectively causing her to ignore the precondition. The corresponding model can be viewed in Fig. 6. Here, action point $\textbf{np}$ corresponds to ‘agent *a* truthfully states that $\lnot B_a p$’, whereas action point $\textbf{p}$ corresponds to ‘agent *a* successfully lies that $\lnot B_a p$.’

Applying the action model in Fig. 6 to the model in Fig. 3 yields an $M^{\prime },s^{\prime }$ where $$M' = M \otimes \textbf{M} = (S',R',V')$$ and $$s' = (t_1, \textbf{p})$$. The resulting set of states is $$S' = S\times \textbf{S}=\{(t_1,\textbf{np}),(t_2,\textbf{np}),(u_1,\textbf{np}),(u_2,\textbf{np}),(t_1,\textbf{p}),(t_2,\textbf{p}),(u_1,\textbf{p}),(u_2,\textbf{p})\}$$. If an atom is true in a state $$s_1$$, it will be true in $$(s_1,\textbf{x})$$, for any $$\textbf{x}$$. Therefore, $$V'(p) = \{(t_1,\textbf{np}),(u_1,\textbf{np}),(t_1,\textbf{p}),(u_1,\textbf{p})\}$$.

Agent *a* at ToM-1 has $\lnot B_ap$ and $B_ap$ as preconditions. Her relations are $R'(a,1)=\{((t_2,\textbf{np}),(t_2,\textbf{np})),((t_1,\textbf{p}),(t_1,\textbf{p}))\}$. Agent *b* at ToM-0 uses ⊤ as preconditions for both action points, so her relations are the product of *R*(*b*, 0) and $\textbf{R}(b)$, which is $\{((t_1,\textbf{np}),(u_1,\textbf{np})),((t_1,\textbf{np})),\,\,(u_2,\textbf{np})),\,\,((t_2,\textbf{np}),\,(u_1,\textbf{np})),\,((t_2,\textbf{np}),\,(u_2,\textbf{np})),$ $((u_1,\textbf{np}),\,\,(u_1,\textbf{np})),((u_1,\textbf{np}),(u_2,\textbf{np})),((u_2,\textbf{np}),\,(u_1,\textbf{np})),\,((u_2,\textbf{np}),\,(u_2,\textbf{np})),$ $((t_1,\textbf{p}),\,\,(u_1,\textbf{np})),\,\,((t_1,\textbf{p})),\,\,(u_2,\,\,\textbf{np})), \,\, ((t_2,\textbf{p}),\,\,(u_1,\,\,\textbf{np})),\,\,((t_2,\,\,\textbf{p}),\,\,(u_2,\,\,\textbf{np})),$ $((u_1,\textbf{p}),(u_1,\textbf{np})),((u_1,\textbf{p}),(u_2,\textbf{np})), ((u_2,\textbf{p}),(u_1,\textbf{np})),((u_2,\textbf{p}),(u_2,\textbf{np}))\}$.

**Fig. 7:**
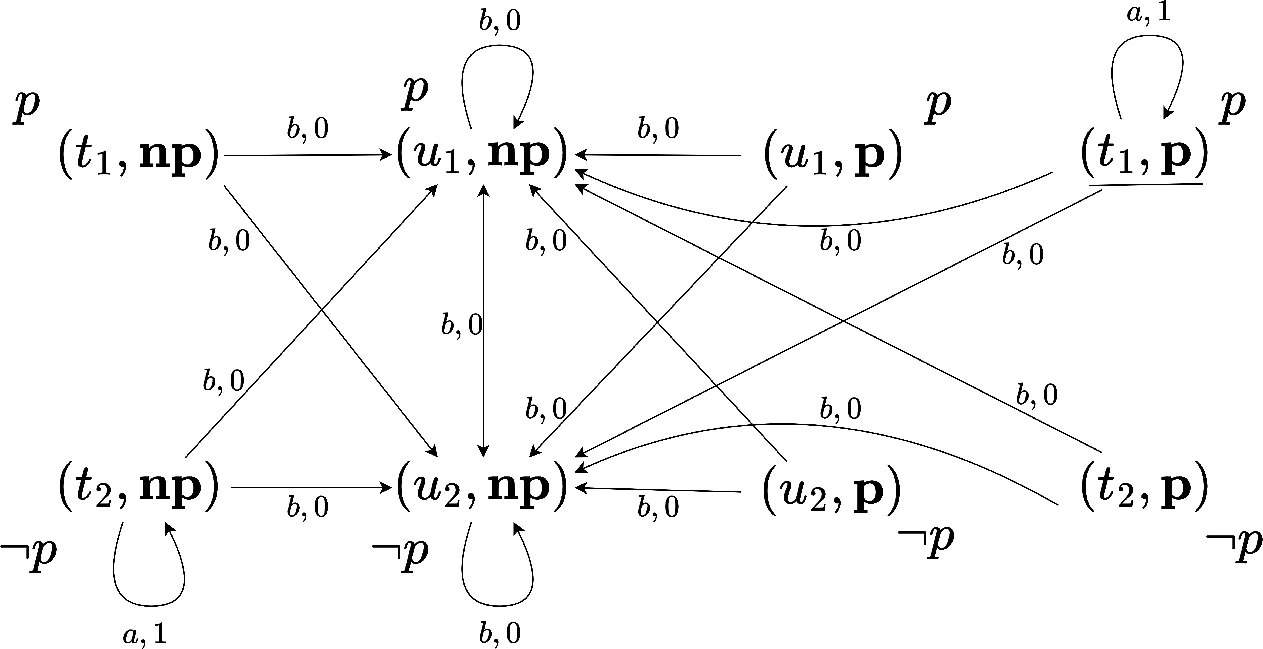
The resulting model after applying the action where agent *a* lies that $\lnot B_ap$, to the model where *a* knows that *p* and *b* does not. Agent *a* (still) uses ToM-1, and agent *b* (still) uses ToM-0. The actual state has been underlined.

The resulting epistemic state $M', (t_1,\textbf{p})$ can be found in Fig. 7.

As seen in Fig. 7, after agent *a* lied to agent *b* that $\lnot B_ap$, agent *b* only considers states $(u_1,\textbf{np})$ and $(u_2,\textbf{np})$ to be possible. She cannot distinguish between these states, so she did not learn whether *p*, as her ToM order is too low to understand the lie. Note here that agent *a* does not consider states $(t_1,\textbf{np})$ or $(t_2,\textbf{p})$ to be possible. The state $(t_1,\textbf{np})$ corresponds to a state where *p* is true, yet agent *a* truthfully states that $\lnot B_a p$. State $(t_2,\textbf{p})$ corresponds to a state where *p* is false, yet agent *a* lies that $\lnot B_a p$. As seen in Fig. 3, agent *a* already knows that *p* in $t_1$, so she cannot truthfully state that $\lnot B_a p$ in $t_1$. Similarly, in $t_2$ she already knows that ¬p, so she cannot lie that $\lnot B_a p$.

We can also model how agent *a* lies that she believes that *p* is false. This is done by replacing the precondition of $\textbf{np}$ with $B_a\lnot p$. The resulting model is *also* the model found in Fig. 7.

In this particular example, we can remove states $(t_1,\textbf{np})$, $(u_1,\textbf{p})$, $(u_2,\textbf{p})$, and $(t_2,\textbf{p})$ without affecting the truth value of formulas at state $(t_1,\textbf{p})$. None of those states have incoming relations, so they cannot be reached using belief operators. However, if we initially used epistemic state $M, u_1$ instead of $M, t_1$, then the update would result in epistemic state $M', (u_1,\textbf{p})$. We cannot remove states simply because there are no incoming relations. A good example of this is the epistemic state $M, s_1$ with M=(S,R,V) where $S=\{s_1,s_2,s_3\}$, $R(a,0) = \{(s_1,s_2),(s_2,s_2)\}$, and $R(b,0) = \{(s_1,s_3),(s_3,s_3)\}$. In this example, both agents *a* and *b* use ToM-0, and neither agent considers state $s_1$ to be possible.

#### An example of actions and decreasing theory of mind orders

We now show an example of applying a public announcement to a model with decreasing ToM orders. Consider once again the epistemic state $M,s_1$ shown in Fig. 5 of Section 2.7.2.

**Fig. 8:**
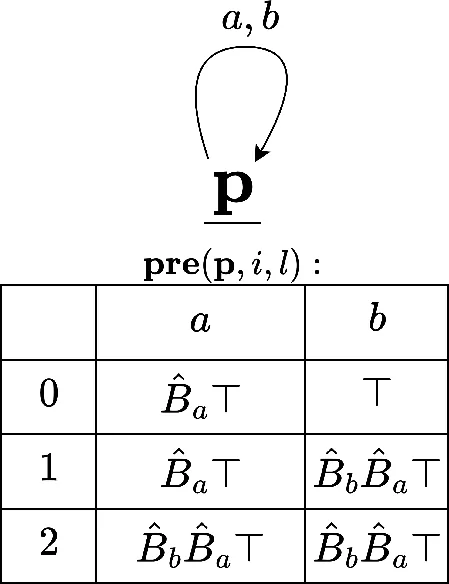
A pointed action where agent *b* truthfully and publicly announces ‘I consider it possible that agent *a* considers it possible that ⊤’. Agents cut belief operators from left to right until they can understand the announcement given their ToM order.

We resolve an action that models agent *b* truthfully and publicly announcing that she considers it possible that agent *a* considers it possible that ⊤, which corresponds to the formula $\hat{B}_b\hat{B}_a\top$ being announced. A public announcement is a special single-action action model with a reflexive relation for all agents. Here, the precondition for the only action point is the formula that is being announced. In this example, we follow the methods of Minculescu (2024) by having agents cut belief operators, left to right, from announcements they do not understand. For agent *a*, the announcement $\hat{B}_b\hat{B}_a\top$ requires two perspective switches (from *a* to *b* and then back to *a*), whereas for agent *b*, it only requires one perspective switch. The action model is a pointed action $(\textbf{M},\textbf{p})$ with $\textbf{M} = (\textbf{S},\textbf{R},\textbf{pre})$ and $\textbf{p}\in \textbf{S}$. It has action points $\textbf{S}=\{\textbf{p}\}$, relations $\textbf{R}(a)=\textbf{R}(b)=\{\textbf{p},\textbf{p}\}$, and preconditions $\textbf{pre}(\textbf{p},a,2) = \textbf{pre}(\textbf{p},b,2) = \textbf{pre}(\textbf{p},b,1) = \hat{B}_b\hat{B}_a\top$, $\textbf{pre}(\textbf{p},a,1) = \textbf{pre}(\textbf{p},a,0) = \hat{B}_a\top$, and $\textbf{pre}(\textbf{p},b,0) = \top$. Here, agents perceive the announcement as the formula obtained by cutting belief operators from left to right until it matches their ToM order. The corresponding action model can be found in Fig. 8.

**Fig. 9:**
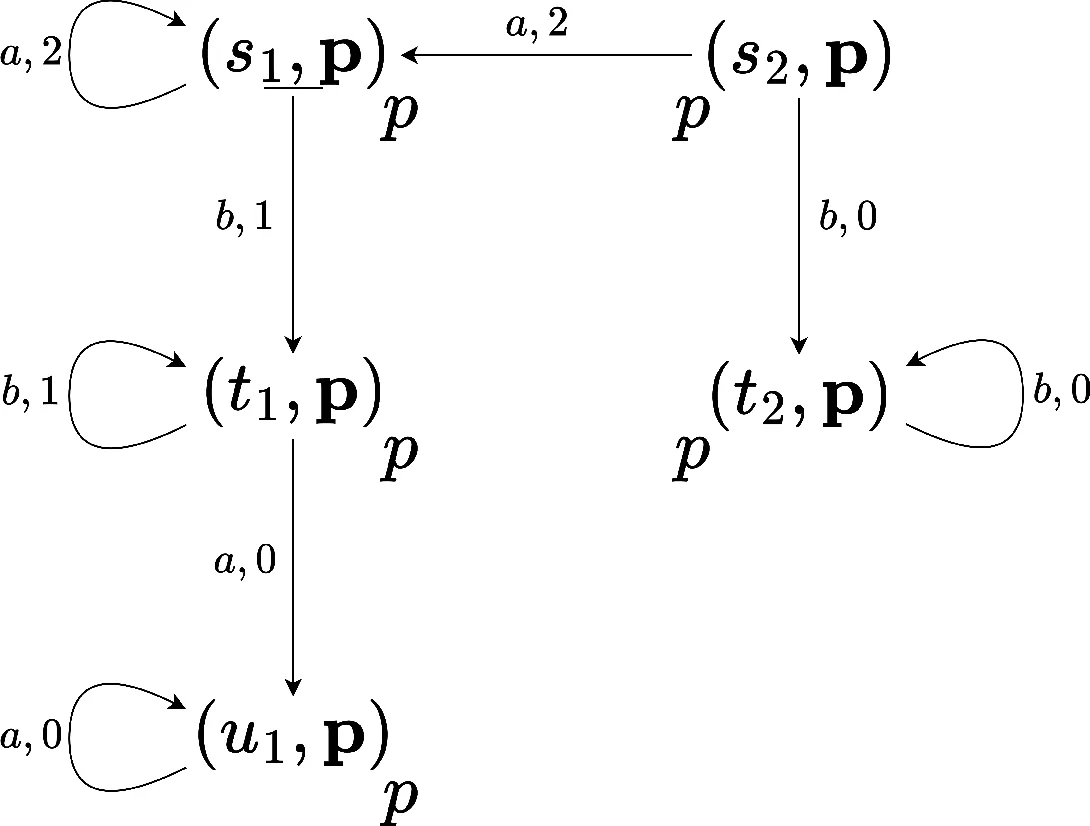
The resulting model after applying the truthful public announcement of $\hat{B}_b\hat{B}_a\top$ to the model where *a* is uncertain about agent *b*’s ToM order. In the new model, agent *a* no longer considers state $s_2$ (now $(s_2,\textbf{p})$) to be possible.

Applying the action model in Fig. 8 to the model in Fig. 5 yields an $M^{\prime },s^{\prime }$ where $$M' = M\otimes \textbf{M}=(S',R',V')$$ and $$s' = (s_1,\textbf{p})$$. The resulting set of states is $$S' = S\times \textbf{S} = \{(s_1,\textbf{p}), (s_2,\textbf{p}), (t_1,\textbf{p}), (t_2,\textbf{p}), (u_1,\textbf{p})\}$$ and the valuation is $$V'(p) = S\times \textbf{S}$$. In $$s_2$$, the announcement $$\hat{B}_b\hat{B}_a\top $$ is false, so agent *a*, at ToM order 2, no longer considers $$s_2$$ (now $$(s_2,\textbf{s})$$) to be possible. All other relations stay the same. The resulting relations are $$R'(a,2) = \{((s_1,\textbf{p}),(s_1,\textbf{p})),((s_2,\textbf{p}),(s_1,\textbf{p}))\}$$, $$R'(a,0) = \{((t_1,\textbf{p}),(u_1,\textbf{p})),((u_1,\textbf{p}),(u_1,\textbf{p}))\}$$, $$R'(b,1)=\{((s_1,\textbf{p}),(t_1,\textbf{p})),((t_1,\textbf{p}),(t_1,\textbf{p}))\}$$, and $$R'(b,0)=\{((s_2,\textbf{p}),(t_2,\textbf{p})),((t_2,\textbf{p}),(t_2,\textbf{p}))\}$$. The resulting epistemic state $$M',(s_1,\textbf{p})$$ can be found in Fig. 9.

In Fig. 9, it can be seen that agent *a* now believes that agent *b* believes that agent *a* considers it possible that ⊤. That is, $B_aB_b\hat{B}_a\top$. Agent *a* is now certain that agent *b* is at least a first-order ToM user. Agent *b* announcing that she considers it possible that *a* considers it possible that ⊤ indeed required *b* to make a first-order ToM attribution.

Note that we obtain the same result if we use $\textbf{pre}(\textbf{p},a,0) = \textbf{pre}(\textbf{p},a,1) = \top$. In this particular example, cutting belief operators from preconditions whenever their ToM order exceeds that of the agents, yields the same result as replacing those preconditions with ⊤.

### Axioms for actions

To add actions to our previous axiom systems, we propose the following reduction axioms, which take inspiration from van Ditmarsch et al. (2007):

#### Definition 14

(*Axioms of actions*)

$$\begin{array}{|l|l|l|} \hline \text {AM1}& \text {Atomic Permanence}& [\textbf{M},\textbf{s}]p\leftrightarrow p\\ \hline \text {AM2}& \text {Action and Negation}& [\textbf{M},\textbf{s}]\lnot \varphi \leftrightarrow \lnot [\textbf{M},\textbf{s}]\varphi \\ \hline \text {AM3}& \text {Action and Conjunction}& [\textbf{M},\textbf{s}](\varphi \wedge \psi )\leftrightarrow ([\textbf{M},\textbf{s}]\varphi \wedge [\textbf{M},\textbf{s}]\psi )\\ \hline \text {AM4}& \text {Action and Beliefs}& [\textbf{M},\textbf{s}]B_i^l\varphi \leftrightarrow \bigwedge _{(\textbf{s},\textbf{t})\in \textbf{R}(i)}B_i^l(\textbf{pre}(\textbf{t},i,l)\rightarrow [\textbf{M},\textbf{t}]\varphi )\\ \hline \text {AM5}& \text {Action Composition}& [\textbf{M},\textbf{s}][\mathbf {M'},\textbf{s}']\varphi \leftrightarrow [\textbf{M},\textbf{s};\textbf{M}',\textbf{s}']\varphi \\ \hline \end{array}$$

Axiom systems ToMea, ToMa, and ToM45a are obtained by adding our action axioms to the systems ToMe, ToM, and ToM45, respectively. For axiom AM5, we must define action composition:

#### Definition 15

(*Action Composition*) - Let $\textbf{M} = (\textbf{S},\textbf{R},\textbf{pre})$ and $\textbf{M}' = (\textbf{S}',\textbf{R}',\textbf{pre}')$. Their composition $\textbf{M};\textbf{M}'$ is the action model $(\textbf{S}'',\textbf{R}'',\textbf{pre}'')$ such that

$$ \begin{array}{lll} \textbf{S}''& =& \textbf{S}\times \textbf{S}'\\ ((\textbf{s},\textbf{s}'),(\textbf{t},\textbf{t}'))\in \textbf{R}''(i)& \text {iff}& (\textbf{s},\textbf{t})\in \textbf{R}(i)\ and\ (\textbf{s}',\textbf{t}')\in \textbf{R}'(i)\\ \textbf{pre}''((\textbf{s},\textbf{s}'),i,l)& =& \textbf{pre}(\textbf{s},i,l)\wedge [\textbf{M},\textbf{s}]\textbf{pre}'(\textbf{s}',i,l).\\ \end{array} $$

Axiom AM4 is somewhat involved, so consider a simple example. Take the formula $[\textbf{M},\mathbf {s_1}]B_a^0p$ with $\textbf{M} = (\textbf{S},\textbf{R},\textbf{pre})$ where $\textbf{S}=\{\mathbf {s_1},\mathbf {s_2}\}$, $\textbf{R}(a)=\{(\mathbf {s_1},\mathbf {s_1}),(\mathbf {s_1},\mathbf {s_2})\}$, $\textbf{pre}(\mathbf {s_1},a,0) = p$, and $\textbf{pre}(\mathbf {s_2},a,0) = B_b p$. Axiom AM4 states this formula is equivalent to a conjunction over all action points that agent *a* considers possible from $\mathbf {s_1}$. In our example, this is the formula $B_a^0(\textbf{pre}(\mathbf {s_1},a,0)\rightarrow [\textbf{M},\mathbf {s_1}]p)\wedge B_a^0(\textbf{pre}(\mathbf {s_2},a,0)\rightarrow [\textbf{M},\mathbf {s_2}]p)$, which is $B_a^0(p\rightarrow [\textbf{M},\mathbf {s_1}]p)\wedge B_a^0(B_b p\rightarrow [\textbf{M},\mathbf {s_2}]p)$.

### Soundness of system ToMea

We now show soundness and strong completeness for ToMea, from which soundness and strong completeness of ToMa and ToM45a can readily be constructed. Once again, we allow for possibly infinite sets of premises Δ. First, we show soundness of axioms AM4 and AM5 in Lemma 3 and Lemma 4, respectively. Afterwards, we show soundness of system ToMea in Theorem 2.

#### Lemma 3

Axiom AM4 is valid. That is,

$$\begin{aligned} \begin{array}{c}\models [\textbf{M},\textbf{s}]B_i^l\varphi \leftrightarrow \bigwedge _{(\textbf{s},\textbf{t})\in \textbf{R}(i)}B_i^l(\textbf{pre}(\textbf{t},i,l)\rightarrow [\textbf{M},\textbf{t}]\varphi )\end{array} \end{aligned}$$

#### Proof

First, noting that $[\textbf{M},\textbf{s}]\varphi \Leftrightarrow \lnot [\textbf{M},\textbf{s}]\lnot \varphi$, we rewrite the formula to

$$\begin{aligned} \begin{array}{c}\models [\textbf{M},\textbf{s}]\hat{B}_i^l\varphi \leftrightarrow \bigvee _{(\textbf{s},\textbf{t})\in \textbf{R}(i)}\hat{B}_i^l(\textbf{pre}(\textbf{t},i,l)\wedge [\textbf{M},\textbf{t}]\varphi )\end{array} \end{aligned}$$

by taking the dual of both sides of the biconditional. Next, we prove both directions of the biconditional separately. Assume an arbitrary *M*, *s*.

**Left to right. **Assume $$M,s\models [\textbf{M},\textbf{s}]\hat{B}_i^l\varphi $$. Then, by definition of actions, $$(M\otimes \textbf{M},(s,\textbf{s}))\models \hat{B}_i^l\varphi .$$ We use $M^{\prime }=\left(S^{\prime },R^{\prime },V^{\prime }\right)$ for $$M\otimes \textbf{M}$$. Since $$\hat{B}_i^l\varphi $$ holds at $$(s,\textbf{s})$$ there must be a $$(t,\textbf{t})\in S'$$ with $$((s,\textbf{s}),(t,\textbf{t}))\in R'(i,l)$$ and $$(M\otimes \textbf{M},(t,\textbf{t}))\models \varphi $$. From the latter, we have that $$M,t\models [\textbf{M},\textbf{t}]\varphi $$. As we have $$((s,\textbf{s}),(t,\textbf{t}))\in R'(i,l)$$ it must be the case, from the definition of $R^{\prime }$, that $$(\textbf{s},\textbf{t})\in \textbf{R}(i)$$, (s,t)∈R(i,l), and $$M,t\models \textbf{pre}(\textbf{t},i,l)$$. Then also $$M,t\models \textbf{pre}(\textbf{t},i,l)\wedge [\textbf{M},\textbf{t}]\varphi $$, and from (s,t)∈R(i,l) it follows that $$M,s\models \hat{B}_i^l(\textbf{pre}(\textbf{t},i,l)\wedge [\textbf{M},\textbf{t}]\varphi )$$, where $$\textbf{t}$$ is just some action point with $$(\textbf{s},\textbf{t})\in \textbf{R}(i)$$. From this it immediately follows that $$M,s\models \bigvee _{(\textbf{s},\textbf{t})\in \textbf{R}(i)} \hat{B}_i^l(\textbf{pre}(\textbf{t},i,l)\wedge [\textbf{M},\textbf{t}]\varphi )$$, which is what we set out to show.

**Right to left. **Assume $M,s\models \bigvee _{(\textbf{s},\textbf{t})\in \textbf{R}(i)}\hat{B}_i^l(\textbf{pre}(\textbf{t},i,l)\wedge [\textbf{M},\textbf{t}]\varphi )$. Then there is some $\textbf{t}$ with $(\textbf{s},\textbf{t})\in \textbf{R}(i)$ and $M,s\models \hat{B}_i^l(\textbf{pre}(\textbf{t},i,l)\wedge [\textbf{M},\textbf{t}]\varphi )$. From this it follows that there is a t∈S such that (s,t)∈R(i,l) with $M,t\models \textbf{pre}(\textbf{t},i,l)$ and $M,t\models [\textbf{M},\textbf{t}]\varphi$. From the latter it holds that $(M\otimes \textbf{M},(t,\textbf{t}))\models \varphi$. From (s,t)∈R(i,l), $(\textbf{s},\textbf{t})\in \textbf{R}(i)$, and $M,t\models \textbf{pre}(\textbf{t},i,l)$, it follows that $((s,\textbf{s}),(t,\textbf{t}))\in R'(i,l)$ (here, $M\otimes \textbf{M} = (S',R',V')$). Given that $(M\otimes \textbf{M},(t,\textbf{t}))\models \varphi$, we now have that $(M\otimes \textbf{M},(s,\textbf{s}))\models \hat{B}_i^l\varphi$, from which it follows that $M,s\models [\textbf{M},\textbf{s}]\hat{B}_i^l\varphi$, which is exactly what we set out to show.

**Conclusion. **Combining the right-to-left and left-to-right directions we obtain $\models [\textbf{M},\textbf{s}]\hat{B}_i^l\varphi \leftrightarrow \bigvee _{(\textbf{s},\textbf{t})\in \textbf{R}(i)}\hat{B}_i^l(\textbf{pre}(\textbf{t},i,l)\wedge [\textbf{M},\textbf{t}]\varphi )$, from which it immediately follows that $\models [\textbf{M},\textbf{s}]B_i^l\varphi \leftrightarrow \bigwedge _{(\textbf{s},\textbf{t})\in \textbf{R}(i)}B_i^l(\textbf{pre}(\textbf{t},i,l)\rightarrow [\textbf{M},\textbf{t}]\varphi )$. □

Next, we show that axiom AM5 is valid.

#### Lemma 4

Axiom AM5 is valid. That is, $\models [\textbf{M},\textbf{s}][\textbf{M}',\textbf{s}']\varphi \leftrightarrow [\textbf{M},\textbf{s};\textbf{M}',\textbf{s}']\varphi$.

#### Proof

Take two arbitrary actions $(\textbf{M},\textbf{s})$ and $(\textbf{M}',\textbf{s}')$, an arbitrary formula φ, and an arbitrary epistemic state *M*, *s*. We must show that $[\textbf{M},\textbf{s}][\textbf{M}',\textbf{s}']\varphi$ is equivalent to $[\textbf{M},\textbf{s};\textbf{M}',\textbf{s}']\varphi$. We do this by showing that $((M\otimes \textbf{M})\otimes \textbf{M}')$ is isomorphic to $(M\otimes (\textbf{M};\textbf{M}'))$. The states, valuation, and relations are covered separately. For the remainder of this proof, we define the *domain* $\mathcal {D}(M_a)$ of a model $M_a=(S_a,R_a,V_a)$ to be its set of states $S_a$, and similarly for action models.

**States - **Suppose we have that $(s,(\textbf{s},\textbf{s}'))\in \mathcal {D}(M\otimes (\textbf{M};\textbf{M}'))$. Then also s∈S and $(\textbf{s},\textbf{s}')\in \mathcal {D}(\textbf{M};\textbf{M}')$. From the latter we have that $\textbf{s}\in \textbf{S}$ and $\mathbf {s'}\in \textbf{S}'$. Now we obtain that $(s,\textbf{s})\in \mathcal {D}(M\otimes \textbf{M})$, and from there we obtain that $((s,\textbf{s}),\textbf{s}')\in \mathcal {D}((M\otimes \textbf{M})\otimes \textbf{M}')$. Running the argument in reverse yields the other direction.

**Valuation - **Analogous to states.

**Relations - **Take any t∈S, $\textbf{t}\in \textbf{S}$, and $\textbf{t}'\in \textbf{S}'$. We must show: $((s,(\textbf{s},\textbf{s}')),(t,(\textbf{t},\textbf{t}')))\in R_{M\otimes (\textbf{M};\textbf{M}')}(i,l)\Leftrightarrow (((s,\textbf{s}),\textbf{s}'),((t,\textbf{t}),\textbf{t}'))\in R_{(M\otimes \textbf{M})\otimes \textbf{M}'}(i,l)$. We show the right-to-left direction, leaving left-to-right to the reader.

**Right to left - **For readability, we number our proof steps.

$$ \begin{array}{llll} (1)& \text {Suppose }& (((s,\textbf{s}),\textbf{s}'),((t,\textbf{t}),\textbf{t}'))\in R_{(M\otimes \textbf{M})\otimes \textbf{M}'}(i,l).& \\ (2)& \text {Then also }& ((s,\textbf{s}),(t,\textbf{t}))\in R_{(M\otimes \textbf{M})}(i,l),& (\text {from }(1))\\ (3)& & (\textbf{s}',\textbf{t}')\in \textbf{R}'(i),& (\text {from }(1))\\ (4)& \text {and}& M\otimes \textbf{M}, (t,\textbf{t})\models \textbf{pre}'(\textbf{t}',i,l).& (\text {from }(1))\\ (5)& \text {Furthermore, }& (s,t)\in R(i,l),& (\text {from }(2))\\ (6)& & (\textbf{s},\textbf{t})\in \textbf{R}(i),& (\text {from }(2))\\ (7)& \text {and }& M,t\models \textbf{pre}(\textbf{t},i,l).& (\text {from }(2))\\ (8)& \text {Next, we know}& M,t\models [\textbf{M},\textbf{t}]\textbf{pre}'(\textbf{t}',i,l),& (\text {from (4)})\\ (9)& \text {which gives us}& M,t\models \textbf{pre}(\textbf{t},i,l)\wedge [\textbf{M},\textbf{t}]\textbf{pre}'(\textbf{t}',i,l).& (\text {from }(7)\text { and }(8))\\ (10)& \text {Furthermore,}& ((\textbf{s},\mathbf {s'}),(\textbf{t},\textbf{t}'))\in \textbf{R}_{(\textbf{M};\textbf{M})}(i),& (\text {from }(6)\text { and }(3))\\ (11)& \text {so we conclude}& ((s,(\textbf{s},\textbf{s}')),(t,(\textbf{t},\textbf{t}')))\in R_{M\otimes (\textbf{M};\textbf{M}')}(i,l).& (\text {from (5), (10) and (9)})\\ \end{array} $$

**Left to right - **This direction is left to the reader; it uses similar techniques.

**Conclusion - **By proving isomorphism for the states, valuation, and relations, we have shown that $((M\otimes \textbf{M})\otimes \textbf{M}')$ is isomorphic to $(M\otimes (\textbf{M};\textbf{M}'))$, and therefore that $\models [\textbf{M},\textbf{s}][\textbf{M}',\textbf{s}']\varphi \leftrightarrow [\textbf{M},\textbf{s};\textbf{M}',\textbf{s}']\varphi$. □

Finally, we show soundness of ToMea.

#### Theorem 2

Axiom system ToMea is sound with respect to its semantics.

#### Proof

To show soundness of ToMea, we show that $\Delta \vdash \varphi \Rightarrow \Delta \models \varphi$ for all (possibly infinite) sets of premises Δ. We have soundness of ToMe, so we only show soundness of axioms AM1 through AM5. Above, we have shown soundness of AM4 and AM5 in Lemma 3 and Lemma 4, respectively. Soundness of the other axioms is left to the reader: Soundness of AM1, AM2, and AM3 can be proven either directly or in very few steps from our semantic definitions. Having shown soundness for ToMe with Theorem 1, as well as soundness for axioms AM1 through AM5, we conclude, through an inductive proof over the derivations in ToMea, that the axiom system of ToMea is sound with respect to its semantics. □

### Strong completeness of system ToMea

To show strong completeness of ToMea we use a *proof by translation*, a standard method when showing completeness of dynamic logics (Kooi, 2007; van Ditmarsch et al., 2007; Baltag et al., 2023). We define a translation function t(φ) that translates any formula φ into an equivalent one that does not use any action operators, and then proceed to show that $\vdash \varphi \leftrightarrow t(\varphi )$, and hence also $\Delta \vdash \varphi \leftrightarrow t(\varphi )$ for all (possibly infinite) sets of premises Δ. To be able to use a proof by induction for the latter, we define our own complexity measure c(φ) such that the ordering of the complexity of formulas corresponds to the ordering inherent in our translation function. First, we define our translation function t(φ), for all formulas φ of ToMea, as follows:

#### Definition 16

The translation function t(φ) of formulas φ, inspired by van Ditmarsch et al. (2007), is defined as

$$ \begin{array}{lll} t(p)& =& p\\ t(\lnot \varphi )& =& \lnot t(\varphi )\\ t(\varphi \wedge \psi )& =& t(\varphi )\wedge t(\psi )\\ t(B_i^l\varphi )& =& B_i^l t(\varphi )\\ t([\textbf{M},\textbf{s}]p)& =& t(p)\\ t([\textbf{M},\textbf{s}]\lnot \varphi )& =& t(\lnot [\textbf{M},\textbf{s}]\varphi )\\ t([\textbf{M},\textbf{s}](\varphi \wedge \psi )& =& t[\textbf{M},\textbf{s}]\varphi )\wedge ([\textbf{M},\textbf{s}]\psi )\\ t([\textbf{M},\textbf{s}] B_i^l\varphi )& =& t(\bigwedge _{(\textbf{s},\textbf{t})\in \textbf{R}(i)}B_i^l(\textbf{pre}(\textbf{t},i,l)\rightarrow [\textbf{M},\textbf{t}]\varphi ))\\ t([\textbf{M},\textbf{s}][\mathbf {M'},\textbf{s}']\varphi )& =& t([\textbf{M},\textbf{s};\textbf{M}',\textbf{s}']\varphi )\\ \end{array} $$

As is usual in such translation functions, in the formulas on the right-hand side, the action operator has been pushed inwards. Furthermore, our translation corresponds exactly to our axiom system. Next, we define the complexity measure we use in our inductive proof of $\vdash \varphi \leftrightarrow t(\varphi )$, which is inspired by van Ditmarsch et al. (2007)^4^.

#### Definition 17

*Complexity*

We define the complexity c(φ) of a formula φ by induction, as follows:

$$ \begin{array}{lll} c(p)& =& 1\\ c(\lnot \varphi )& =& 1+c(\varphi )\\ c(\varphi \wedge \psi )& =& 1+max(\{c(\varphi ),c(\psi )\})\\ c(B_i^l\varphi )& =& 1+c(\varphi )\\ c([\textbf{M},\textbf{s}]\varphi )& =& {(3+c(\textbf{M}))\cdot c(\varphi )}\\ {c(\textbf{M})}& =& |\textbf{R}| + max\{c(\textbf{pre}(\textbf{t},i,l))|\textbf{t}\in \textbf{M},i\in A,l\in L\}\\ \end{array} $$

We may omit ‘$i\in A, l\in L$’ in the complexity of $\textbf{M}$. Here, $|\textbf{R}|$ is the cardinality of the relations of action model $\textbf{M}=(\textbf{S},\textbf{R},\textbf{pre})$, or the total number of relations $(\textbf{s},\textbf{t})\in \textbf{R}(i)$ for any $\textbf{s},\textbf{t}\in \textbf{S}$ across all i∈A. Furthermore, $\textbf{t}\in \textbf{M}$ is shorthand for ‘those $\textbf{t}\in \textbf{S}$ where $\textbf{M}=(\textbf{S},\textbf{R},\textbf{pre})$’. Note that we assume the complexity of a pointed action model $(\textbf{M},\textbf{s})$ to be the same as the complexity of a non-pointed action model $\textbf{M}$, because the preconditions of all action points of $\textbf{M}$ are taken into account for $c(\textbf{M})$, including $\textbf{s}$ itself. The complexity of $c([\textbf{M},\textbf{s}]\varphi )$ does not depend on the chosen $\textbf{s}$.

#### Lemma 5

The complexity measure in Definition 17 has the following properties:

1. $$\text {if }\varphi \in Sub(\psi )\text {, then }c(\psi )\ge c(\varphi )$$
2. $$c([\textbf{M},\textbf{s}]p)>c(p)$$
3. $$c([\textbf{M},\textbf{s}]\lnot \varphi ) > c(\lnot [\textbf{M},\textbf{s}]\varphi )$$
4. $$c([\textbf{M},\textbf{s}](\varphi \wedge \psi )) > c([\textbf{M},\textbf{s}]\varphi \wedge [\textbf{M},\textbf{s}]\psi )$$
5. $$c([\textbf{M},\textbf{s}] B_i^l\varphi ) > c\left( \bigwedge _{(\textbf{s},\textbf{t})\in \textbf{R}(i)}B_i^l(\textbf{pre}(\textbf{t},i,l)\rightarrow [\textbf{M},\textbf{t}]\varphi )\right) $$
6. $$c([\textbf{M},\textbf{s}][\mathbf {M'},\textbf{s}']\varphi ) > c([\textbf{M},\textbf{s};\textbf{M}',\textbf{s}']\varphi )$$

Here, Sub(ψ) is the set of subformulas of ψ, including ψ itself. Subformulas are defined in the usual manner, following Baltag et al. (2016): The subformulas of $[\textbf{M},\textbf{s}]\varphi$ are $[\textbf{M},\textbf{s}]\varphi$ itself, all subformulas of φ, and all subformulas of all preconditions that occur in $\textbf{M}$. Lemma 5 is proven by proving each property separately. We show proofs for items 5 and 6, where the other items are handled akin to the complexity of public announcement logic in van Ditmarsch et al. (2007), and are therefore omitted.

#### Proof of Lemma 5.5

We rewrite both sides of the inequality in turn.

**Left-hand side. ** First, we rewrite the left-hand side using a series of equalities:

$$ \begin{array}{ll} c([\textbf{M},\textbf{s}] B_i^l\varphi )& =\\ (3+c(\textbf{M}))\cdot c(B_i^l\varphi )& =\\ 3+3c(\varphi )+c(\textbf{M})+c(\varphi )c(\textbf{M})& \\ \end{array} $$

**Right-hand side. **Now we rewrite the right-hand side:

$$ \begin{array}{ll} c\left( \bigwedge _{(\textbf{s},\textbf{t})\in \textbf{R}(i)}B_i^l(\textbf{pre}(\textbf{t},i,l)\rightarrow [\textbf{M},\textbf{t}]\varphi )\right) & \\ & =^*\\ n + max\{c(B_i^l(\textbf{pre}(\textbf{t},i,l)\rightarrow [\textbf{M},\textbf{t}]\varphi ))|(\textbf{s},\textbf{t})\in \textbf{R}(i)\}& \\ & =^\dagger \\ n + max\{c(B_i^l\lnot (\lnot \lnot \textbf{pre}(\textbf{t},i,l)\wedge \lnot [\textbf{M},\textbf{t}]\varphi ))|(\textbf{s},\textbf{t})\in \textbf{R}(i)\}& \\ & =\\ 4 + n + max(\{c([\textbf{M},\textbf{t}]\varphi )\}\cup \{1+c(\textbf{pre}(\textbf{t},i,l))|(\textbf{s},\textbf{t})\in \textbf{R}(i)\})& \\ & =^\ddagger \\ 4 + n + c([\textbf{M},\textbf{t}]\varphi )& \\ & =\\ 4+n+3c(\varphi )+c(\varphi )c(\textbf{M})& \\ \end{array} $$

$\phantom {a}^*$ For the outer conjunction, we add 1 for each $(\textbf{s},\textbf{t})\in \textbf{R}(i)$, minus 1. We call this number *n*.

$\phantom {a}^\dagger$ In our syntax, A→B is an abbreviation of $\lnot A\vee B$, which itself is an abbreviation of $\lnot (\lnot \lnot A\wedge \lnot B)$.

$\phantom {a}^\ddagger$ Note that by its definition, $c([\textbf{M},\textbf{t}]\varphi )$ must be at least $1+c(\textbf{pre}(\textbf{t},i,l))$, for any $\textbf{t}\in \textbf{S}$, i∈A, and l∈L.

**Comparison. **From our previous rewrites, we now know we have to show that

$$ \begin{array}{lll} 3+3c(\varphi )+c(\textbf{M})+c(\varphi )c(\textbf{M})& >& 4+n+3c(\varphi )+c(\varphi )c(\textbf{M}).\\ \end{array} $$

After subtracting $3 + 3c(\varphi ) + c(\varphi )c(\textbf{M})$, it remains to show that $c(\textbf{M})>1 + n$, which is the same as showing that $|\textbf{R}|+max\{c(\textbf{pre}(\textbf{t},i,l)|\textbf{t}\in \textbf{M})\}>1+n$. Recall that *n* is the number of $(\textbf{s},\textbf{t})\in \textbf{R}(i)$, minus one. At most, this is $|\textbf{R}|-1$. Therefore, it is sufficient to show that $|\textbf{R}|+max\{c(\textbf{pre}(\textbf{t},i,l)|\textbf{t}\in \textbf{M})\}>|\textbf{R}|$. This holds, because preconditions have at least complexity 1, since preconditions cannot be formulas smaller than atoms. □

Next, we show that item 6 of the ordering of our complexity holds:

#### Proof of Lemma 5.5

We start by rewriting the left-hand side, where we skip any intermediate steps:

$$\begin{array}{ll} c([\textbf{M},\textbf{s}][\mathbf {M'},\textbf{s}']\varphi )& =\\ 9c(\varphi )+3c(\varphi )c(\textbf{M}) + 3c(\varphi )c(\mathbf {M'})+ c(\varphi )c(\textbf{M})c(\mathbf {M'}).& \\ \end{array} $$

Abusing notation, we abbreviate $max\{c(\textbf{pre}(\textbf{t},i,l))|\textbf{t}\in \textbf{M}\}$ to $max(c(\textbf{pre}))$, and $max\{c(\mathbf {pre'}(\mathbf {t'},i,l))|\mathbf {t'}\in \mathbf {M'}\}$ to $max(c(\mathbf {pre'}))$. We continue rewriting to

$$\begin{array}{l}9c(\varphi )+3c(\varphi )\cdot |\textbf{R}|+3c(\varphi )\cdot max(c(\textbf{pre})) + 3c(\varphi )\cdot |\mathbf {R'}| + 3c(\varphi )\cdot max(c(\mathbf {pre'}))+\\ c(\varphi )\cdot \mathbf {|R|}\cdot \mathbf {|R'|} +c(\varphi )\cdot \mathbf {|\textbf{R}|}\cdot max(c(\mathbf {pre'}))+ c(\varphi )\cdot |\mathbf {R'}|\cdot max(c(\textbf{pre}))+\\ c(\varphi )\cdot max(c(\textbf{pre}))\cdot max(c(\mathbf {pre'})).\end{array}$$

Recall from Definition 15 that $\textbf{pre}''((\textbf{s},\textbf{s}'))=\textbf{pre}(\textbf{s})\wedge [\textbf{M},\textbf{s}]\textbf{pre}'(\textbf{s}')$. We now rewrite the right-hand side, where we abbreviate $\textbf{M};\textbf{M}'$ as $\textbf{M}''$.

$$\begin{array}{lr} c([\textbf{M},\textbf{s};\textbf{M}',\textbf{s}']\varphi )& =\\ (3+c(\textbf{M}''))\cdot c(\varphi )& =\\ (3+|\mathbf {R''}|+max\{c(\textbf{pre}(\textbf{t},i,l)\wedge [\textbf{M},\textbf{s}]\textbf{pre}'(\textbf{t}',i,l))|\textbf{t}\in \textbf{M}\text { and }\mathbf {t'}\in \mathbf {M'}\})\cdot c(\varphi )& =\\ (4+|\mathbf {R''}|+max\{(3+c(\textbf{M}))\cdot c(\textbf{pre}'(\textbf{t}',i,l))|\mathbf {t'}\in \mathbf {M'}\})\cdot c(\varphi )& =\\ {4c(\varphi )} + {c(\varphi )\cdot |\mathbf {R''}|} + {3c(\varphi )\cdot max(c(\mathbf {pre'}))} + {c(\varphi )\cdot |\textbf{R}|\cdot max(c(\mathbf {pre'}))} +& \\ {c(\varphi )\cdot max(c(\textbf{pre}))\cdot max(c(\mathbf {pre'}))}& \\ \end{array}$$

Note that the terms ${3c(\varphi )\cdot max(c(\mathbf {pre'}))} + {c(\varphi )\cdot |\textbf{R}|\cdot max(c(\mathbf {pre'}))} + c(\varphi )\cdot max(c(\textbf{pre}))\cdot max(c(\mathbf {pre'}))$ occur in both formulas. If we subtract these terms from the left-hand side, the left-hand side becomes

$$\begin{array}{l}9c(\varphi )+3c(\varphi )\cdot |\textbf{R}|+3c(\varphi )\cdot max(c(\textbf{pre})) + 3c(\varphi )\cdot |\mathbf {R'}| + \\ c(\varphi )\cdot \mathbf {|R|}\cdot \mathbf {|R'|} + c(\varphi )\cdot |\mathbf {R'}|\cdot max(c(\textbf{pre})).\\ \end{array}$$

If we subtract the same terms from the right-hand side, then the right-hand side becomes

$$\begin{aligned} 4c(\varphi ) + {c(\varphi )\cdot |\mathbf {R''}|.} \end{aligned}$$

Therefore, for the left-hand side to be larger than the right-hand side, it is sufficient to show that $9c(\varphi )> 4c(\varphi )$ and $c(\varphi )\cdot |\textbf{R}|\cdot |\textbf{R}'|\ge c(\varphi )\cdot |\textbf{R}''|$. First, note that $9c(\varphi )> 4c(\varphi )$ is immediate. Now we show that $c(\varphi )\cdot \mathbf {|R|}\cdot \mathbf {|R'|} \ge c(\varphi )\cdot |\mathbf {R''}|$. Here, $\mathbf {|R|}\cdot \mathbf {|R'|}\ge |\mathbf {R''}|$ follows directly from the definition of action composition. From this it follows that $c([\textbf{M},\textbf{s}][\mathbf {M'},\textbf{s}']\varphi ) > c([\textbf{M},\textbf{s};\textbf{M}',\textbf{s}']\varphi )$, which is what we set out to prove. □

Now we can proceed to show that $\Delta \vdash \varphi \leftrightarrow t(\varphi )$:

#### Lemma 6

For all formulas φ and all (possibly infinite) sets of premises Δ, the following holds: $\Delta \vdash \varphi \leftrightarrow t(\varphi )$

#### Proof

By induction on c(φ), found in Definition 17, we show that $\vdash \varphi \leftrightarrow t(\varphi )$, from which $\Delta \vdash \varphi \leftrightarrow t(\varphi )$ immediately follows. We follow the proofs in van Ditmarsch et al. (2007).

**Base case - **$\vdash p\leftrightarrow t(p)$. Immediate from the definition of t(φ).

**Inductive hypothesis - **Assume for all φ with $c(\varphi )\le n$ that $\vdash \varphi \leftrightarrow t(\varphi )$.

**Inductive step - **Assume an arbitrary φ with c(φ)=n+1. We proceed with one case for each operator in the language. For formulas $[\textbf{M},\textbf{s}]\varphi$ we include an additional case for each operator in the language. We only show the cases for $\varphi =\lnot \psi$ and $\varphi =[\textbf{M},\textbf{s}](\psi _1\wedge \psi _2)$, leaving the rest to the reader.

**Case** $\lnot \psi$: Then $c(\lnot \psi ) = n + 1$. Therefore, c(ψ)=n, so we can apply the induction hypothesis to ψ, that is, $\vdash \psi \leftrightarrow t(\psi )$. Then by contraposition also $\vdash \lnot \psi \leftrightarrow \lnot t(\psi )$, and by definition of the translation function also $\vdash \lnot \psi \leftrightarrow t(\lnot \psi )$, as required.

**Case** $[\textbf{M},\textbf{s}](\psi _1\wedge \psi _2)$: Then $c([\textbf{M},\textbf{s}](\psi _1\wedge \psi _2)) = n + 1$. But we know from Lemma 5 that $c([\textbf{M},\textbf{s}](\psi _1\wedge \psi _2))>c([\textbf{M},\textbf{s}]\psi _1\wedge [\textbf{M},\textbf{s}]\psi _2)$, so $c([\textbf{M},\textbf{s}]\psi _1\wedge [\textbf{M},\textbf{s}]\psi _2)\le n$. Then the induction hypothesis must hold for $[\textbf{M},\textbf{s}]\psi _1\wedge [\textbf{M},\textbf{s}]\psi _2$. Therefore, $\vdash ([\textbf{M},\textbf{s}]\psi _1\wedge [\textbf{M},\textbf{s}]\psi _2)\leftrightarrow t([\textbf{M},\textbf{s}]\psi _1\wedge [\textbf{M},\textbf{s}]\psi _2)$. Then, from the translation function, we have that $\vdash ([\textbf{M},\textbf{s}]\psi _1\wedge [\textbf{M},\textbf{s}]\psi _2)\leftrightarrow t([\textbf{M},\textbf{s}](\psi _1\wedge \psi _2))$. But from axiom AM3 we also know that $\vdash [\textbf{M},\textbf{s}](\psi _1\wedge \psi _2)\leftrightarrow ([\textbf{M},\textbf{s}]\psi _1\wedge [\textbf{M},\textbf{s}]\psi _2)$, so also $\vdash [\textbf{M},\textbf{s}](\psi _1\wedge \psi _2)\leftrightarrow t([\textbf{M},\textbf{s}](\psi _1\wedge \psi _2))$, as required.

**Conclusion - **By induction on c(φ) we have shown that for all formulas φ of $\mathcal {L}_{\text {ToMa}}$, it holds that $\vdash \varphi \leftrightarrow t(\varphi )$. From this it immediately follows that $\Delta \vdash \varphi \leftrightarrow t(\varphi )$. □

Now we are ready to show strong completeness:

#### Theorem 3

ToMea is strongly complete with respect to its semantics.

#### Proof

From the soundness of ToMea in Theorem 2, and $\Delta \vdash \varphi \leftrightarrow t(\varphi )$, as shown in Lemma 6, it follows that $\Delta \models \varphi \leftrightarrow t(\varphi )$, for all (possibly infinite) sets of premises Δ. We will show that $\Delta \models \varphi \Rightarrow \Delta \vdash \varphi$. Assume that $\Delta \models \varphi$. Then also $\Delta \models t(\varphi )$, as our axioms are sound. As t(φ) does not contain any action operators, it follows that $\Delta \vdash t(\varphi )$, by strong completeness of ToMe. From $\Delta \vdash \varphi \leftrightarrow t(\varphi )$ and $\Delta \vdash t(\varphi )$ it follows that $\Delta \vdash \varphi$, concluding the proof. □

Strong completeness of ToMa and ToM45a follow from the strong completeness of ToMea and the strong completeness of systems ToM and ToM45 as shown in Section 2.6.

### Preservation of properties

In the present section, we show that the properties Downward-ToM-Other, Downward-ToM-Self, ToM-Transitivity, and ToM-Euclidicity are preserved under action model update. The properties Downward-ToM-Other and Downward-ToM-Self are always preserved, regardless of the action model. ToM-Transitivity and ToM-Euclidicity are only preserved if the action model itself is transitive and euclidean, respectively.

In Lemma 7 respectively Lemma 8, we show preservation under update for ToM-Euclidicity and Downward-ToM-Other, leaving ToM-Transitivity and Downward-ToM-Self to the reader. Afterwards, we show in Theorem 4 that all our properties are preserved under certain conditions.

#### Lemma 7

For euclidean action models, ToM-Euclidicity is preserved under update.

#### Proof

Assume a ToM-euclidean model *M* and a euclidean action model $\textbf{M}$. To show this we only need to show that “If $((s,\textbf{s}),(t,\textbf{t}))\in R_{M\otimes \textbf{M}}(i,l)$ and $((s,\textbf{s}),(u,\textbf{u}))\in R_{M\otimes \textbf{M}}(i,m)$ with l≥m, then also $((t,\textbf{t}),(u,\textbf{u}))\in R_{M\otimes \textbf{M}}(i,m)$”.

So assume that $((s,\textbf{s}),(t,\textbf{t}))\in R_{M\otimes \textbf{M}}(i,l)$ and $((s,\textbf{s}),(u,\textbf{u}))\in R_{M\otimes \textbf{M}}(i,m)$ with l≥m. From the former, it follows that (s,t)∈R(i,l), that $(\textbf{s},\textbf{t})\in \textbf{R}(i)$, and that $M,t\models \textbf{pre}(\textbf{t},i,l)$. From $((s,\textbf{s}),(u,\textbf{u}))\in R_{M\otimes \textbf{M}}(i,m)$, it follows that (s,u)∈R(i,m), $(\textbf{s},\textbf{u})\in \textbf{R}(i)$, and $M,u\models \textbf{pre}(\textbf{u},i,m)$. Now, from the ToM-euclidicity of *M*, we have that (t,u)∈R(i,m), and from the euclidicity of $\textbf{M}$, we have that $(\textbf{t},\textbf{u})\in \textbf{R}(i)$. From these two and $M,u\models \textbf{pre}(\textbf{u},i,m)$, it follows that $((t,\textbf{t}),(u,\textbf{u}))\in R_{M\otimes \textbf{M}}(i,m)$, which is what we set out to show. □

Next, we show preservation for Downward-ToM-Other.

#### Lemma 8

Downward-ToM-Other is preserved under update.

#### Proof

Let *M* be a model with the property Downward-ToM-Other, and take an arbitrary action model $\textbf{M}$. We need to show that “There are no $((s,\textbf{s}), (t,\textbf{t})) \in R_{M\otimes \textbf{M}}(i, m)$ and $((t,\textbf{t}), (u,\textbf{u})) \in R_{M\otimes \textbf{M}}(j, l)$ with i≠j and l≥m”.

To this end, let $((s,\textbf{s}), (t,\textbf{t})) \in R_{M\otimes \textbf{M}}(i, m)$ and $((t,\textbf{t}), (u,\textbf{u})) \in R_{M\otimes \textbf{M}}(j, l)$ be relations. To prove our claim, we now only have to show that i≠j implies l<m.

First, assume that i≠j. From $((s,\textbf{s}), (t,\textbf{t})) \in R_{M\otimes \textbf{M}}(i, m)$, it follows that (s,t)∈R(i,m), and from $((t,\textbf{t}), (u,\textbf{u})) \in R_{M\otimes \textbf{M}}(j, l)$, it follows that (t,u)∈R(j,l). From these two, from the assumption that i≠j, and from model *M* having the property Downward-ToM-Other, it follows immediately that l<m, as required. □

Now, we can show that, under the right circumstances, all our properties are preserved under update.

#### Theorem 4

For transitive and euclidean action models, all of the properties ToM-Transitive, ToM-Euclidicean, Downward-ToM-Other, and Downward-ToM-Self are preserved under update.

#### Proof

ToM-Euclidicity and Downward-ToM-Other are shown in Lemma 7 and Lemma 8, respectively. The proofs for ToM-Transitivity and Downward-ToM-Self are left to the reader. The proof for ToM-Transitivity is similar to that of ToM-Euclidicity, under the assumption that the action models are transitive. The proof for Downward-ToM-Self is near-identical to the proof for Downward-ToM-Other. □

An interesting property of Downward-ToM-Other and Downward-ToM-Self is that they are preserved independently of the structure of the action model. If a model has these properties, then no action can remove them.

## Discussion

The present section discusses potential areas of future research. In Section 4.1, we discuss possible behavioural research. In Section 4.2, we consider how the logic can be improved. Next, we suggest potential uses for our logic in computational cognitive modelling in Section 4.3. Lastly, in Section 4.4, we conclude our work.

### Behavioural research

In the development of our logic we make several assumptions about human theory of mind (ToM) reasoning, which lead to testable hypotheses:

In Section 2.3 we discuss why we do not employ special ToM atoms, unlike the logic in Arthaud and Rinard (2023). However, our justification is only an intuitive one, so we suggest that behavioural research be conducted to investigate the prevalence of reasoning about explicit ToM orders.

In Section 2.5.1 we explain that from the perspective of a ToM-*l* agent, ToM-*m* agents with m≥l do not exist. We interpret such ToM-*l* agents as being ‘confused’, and believing that ToM-*m* agents believe in contradictions. However, the results of the child studies in Birch and Bloom (2003) and Ghrear et al. (2021) suggest that ToM-0 users may reason from their own perspective when answering questions about the beliefs of others, as opposed to attributing contradiction. We recommend that more behavioural experiments be conducted to investigate how adult participants reason about statements that are beyond their ToM capabilities.

A major difference between Arthaud and Rinard (2023)’s depth-bounded epistemic logic and our logic of theory of mind is that announcements in Arthaud and Rinard (2023) decrease an agent’s depth. For example, if an agent can use third-order ToM, it will become a zero-order agent after three announcements of ‘agent *a* believes that *p*.’ Meanwhile, actions in our logic do not decrease the ToM order of our accessibility relations, because children seem quite capable of processing multiple sequential ToM-1 (or higher) announcements despite only having ToM-1 themselves (Arslan et al., 2017b). To resolve this difference, future behavioural research can investigate whether adults’ ToM capabilities decrease when exposed to multiple sequential announcements about other agents’ beliefs.

### Formal logic; Modeling choices

In the present paper we present a dynamic logic with ToM limitations that can be used to model deception. We decided to forego issues on decidability and model checking complexity, leaving these for future work instead. In Section 3.1, we define the preconditions of action models as a function $\textbf{pre}:(S\times A\times L)\rightarrow \mathcal {L}$. In Section 3.4 we show two ways in which these preconditions can be used to have agents ‘ignore’ or ‘cut’ formulas. In ongoing work we extend our logic with a precise formalization of the depth of formulas, similar to Arthaud and Rinard (2023), as well as a formally defined cutting function based on the results of Minculescu (2024); Minculescu et al. (2025). It also remains an open question whether and how our action models can be translated into equivalent action models with precondition functions $\textbf{pre}:S\rightarrow \mathcal {L}$ or $\textbf{pre}:(S\times A)\rightarrow \mathcal {L}$.

If it turns out that humans do regularly reason about the exact ToM orders of others, then our logic needs to be extended with depth atoms. If needed, one can add ToM atoms to our logic by adding special depth atoms $t_i^l$ to the language and semantics, using $M,s\models t_i^l$ iff $s\in V(t_i^l)$, and including novel axioms and model properties. The axiom $\lnot (t_i^l\wedge \lnot B_i^m\bot )$ for m>l prevents agents from *using* an order higher than the one they *have*. A unique depth axiom, $\lnot (t_i^l\wedge t_i^m)$ for l≠m, taken from Arthaud and Rinard (2023), ensures agents only have one ToM order. The axiom $\bigvee _{l\in L}t_i^l$ ensures agents have *at least* one ToM order. Here, ToM orders indicate the *maximum* order an agent *can* use. Further details, as well as soundness and completeness, are left for future work.

In the present paper we opt to model ToM limitations only, even though working memory limitations, which interact with ToM, are well-established (Miller, 1956). We also treat all perspective switches between different agents the same, even though the work of van Viegen (2014) shows that a sequence $B_aB_bB_a$ may be harder to interpret than a sequence $B_aB_bB_c$. Such restrictions can be investigated by devising more involved axioms, or by integrating our logic with the work on depth-bounded reasoning (D’Agostino, 2015).

### Computational cognitive models

While our modeling decisions are inspired by prior behavioural research, our logic can also make its own predictions which we aim to test with future behavioural experiments, using an approach similar to Zhang et al. (2021), Minculescu (2024), and Top et al. (2024). All three of these studies propose their own public announcement logic with ToM limitations, which is used at the core of a computational cognitive model of human reasoning. These models are then fit to experimental data: In Zhang et al. (2021) and Top et al. (2024) the game of Aces and Eights is used, while Minculescu (2024) lets participants play variations of Cheryl’s Birthday puzzle.

Going further, our logic could be used to create training agents similar to the ones employed in Veltman et al. (2019). Such agents may improve ToM use and help aid in deception detection.

### Conclusion

In the present paper we present a sound and strongly complete dynamic epistemic logic with lying and theory of mind (ToM) limitations, which is inspired by prior behavioural research. As the example in Section 3.4.1 shows, our logic can be used to model how agents with bounded ToM can lie to each other. As stated in our introduction, our goal was to develop a logic that can model both lying announcements *and* human ToM limitations. In this paper this is exactly what we do, to our knowledge for the first time in the literature.

## Data Availability

Not applicable.

## Footnotes

1 Following Meyer and van der Hoek (1995), a state $s_\Theta$ is the maximally consistent set Θ itself. To make our proofs easier to follow, we use $s_\Theta$ whenever we use its properties in the model (such as $(s_\Theta ,s_\Psi )\in R(i,l)$), whereas we use Θ whenever we use the formulas in Θ (for example, $B_i^l\varphi \in \Theta$].

2 We suggest naming the variant of **ToMa** where the relations in action models have **T**oM **o**rders ‘ToMato’.

3 A reader familiar with action models may wonder why we do not define the semantics of a formula $[\textbf{M},\textbf{s}]\varphi$ using an implication, such as ‘$M,s\models [\textbf{M},\textbf{s}]\varphi \ { \textit{iff} }\ M,s\models \textbf{pre}(\textbf{s}){ \textit{implies} }(M\otimes \textbf{M}, (s,\textbf{s}))\models \varphi$’ [taken from van Ditmarsch et al. (2007)]. This is because the antecedent $\textbf{pre}(\textbf{s})$ ensures the modal flavour of a formula $[\textbf{M},\textbf{s}]\varphi$: The formula $M,s\models [\textbf{M},\textbf{s}]\varphi$ is vacuously true if there is no state corresponding to *s* after the update. However, we do not need to take into account the vacuous case, since there is always an $(s,\textbf{s})$ corresponding to *s*. In this we follow the semantics of Arrow Update Logic (Kooi & Renne, 2011), which instead uses $M,w \models [U]\varphi { \textit{iff} }(M *U), w \models \varphi$.

4 The complexity measure in van Ditmarsch et al. (2007) contains a mistake in the complexity of the action model. In van Ditmarsch et al. (2007), a factor |*S*| is missing in the sum. In our complexity measure, the role of this |*S*| is fulfilled by the factor |*R*|. This mistake has been repaired in van Ditmarsch et al. (2023). This was pointed out to us by Prof. dr. van Ditmarsch.
